# Comparison of glycosyl donors: a supramer approach

**DOI:** 10.3762/bjoc.20.18

**Published:** 2024-01-31

**Authors:** Anna V Orlova, Nelly N Malysheva, Maria V Panova, Nikita M Podvalnyy, Michael G Medvedev, Leonid O Kononov

**Affiliations:** 1 Laboratory of Glycochemistry, N.D. Zelinsky Institute of Organic Chemistry, Moscow, Russian Federation; 2 Theoretical Chemistry Group, N.D. Zelinsky Institute of Organic Chemistry, Moscow, Russian Federation

**Keywords:** concentration, glycosylation, protecting groups, reactivity, sialic acids, stereoselectivity

## Abstract

The development of new methods for chemical glycosylation commonly includes comparison of various glycosyl donors. An attempted comparison of chemical properties of two sialic acid-based thioglycoside glycosyl donors, differing only in the substituent at O-9 (trifluoroacetyl vs chloroacetyl), at different concentrations (0.05 and 0.15 mol·L^−1^) led to mutually excluding conclusions concerning their relative reactivity and selectivity, which prevented us from revealing a possible influence of remote protective groups at O-9 on glycosylation outcome. According to the results of the supramer analysis of the reaction solutions, this issue might be related to the formation of supramers of glycosyl donors differing in structure hence chemical properties. These results seem to imply that comparison of chemical properties of different glycosyl donors may not be as simple and straightforward as it is usually considered.

## Introduction

Glycoconjugates containing sialic acid occur on the surface of all cell types in a variety of organisms. They participate in a broad spectrum of phenomena including virus and bacterial recognition and cellular adhesion [1–11]. The development of effective means for the preparation of α-sialosides through chemical glycosylation (sialylation) received considerable attention since sialo-containing saccharides and conjugates are important for advancing glycobiology [12–13] and glyco-medicine [14–15].

However, the reliable installation of sialic acid residues in oligosaccharides is a rather difficult issue and poor predictability remains characteristic of the sialylation reaction [16–21]. As in other glycosylation reactions [22–29], the result of sialylation is affected by a variety of variables [30–39] including the nature of protective groups on either partner [26,38,40–45] and concentration of reagents [31,33–34,37,39,43–44,46].

**Scheme 1:**
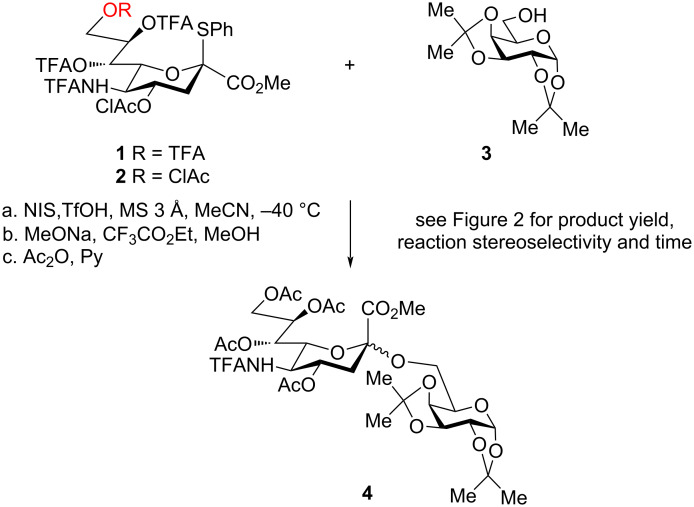
Model sialylation reaction. TFA = CF_3_CO; ClAc = ClCH_2_CO.

During a study of a possible influence of remote acyl protective groups [23] on the sialylation outcome (which could become possible through participation in stabilization of glycosyl cation [24,47–48] in a conformation with all-axial substituents in the pyranose ring [49–53]), we needed to compare two different sialyl donors **1** [36] and **2** with trifluoroacetyl (TFA) and chloroacetyl (ClAc) groups at O-9, respectively, all other substituents being the same. Here, we report the unexpected problems encountered when comparing these glycosyl donors in the sialylation of the primary hydroxy group of the same galactose derivative **3** [54] (see Scheme 1), which eventually led to unprecedented conclusions concerning the very possibility of comparison of chemical properties of different glycosyl donors.

## Results

### Synthesis of glycosyl donor **2**

**Scheme 2:**
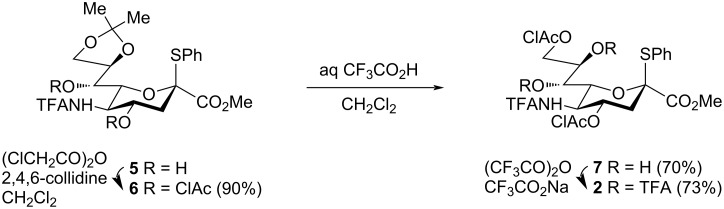
Synthesis of sialyl donor **2**.

Sialyl donor **2** was prepared from the known sialic acid derivative **5** [36] with an 8,9-*O*-isopropylidene group by a three-step reaction sequence (see Scheme 2). Exhaustive chloroacetylation of hydroxy groups in diol **5** with chloroacetic anhydride and 2,4,6-collidine in CH_2_Cl_2_ gave bis-chloroacetate **6** (90% yield), which was treated with 90% aq trifluoroacetic acid in CH_2_Cl_2_ to give diol **7** (70% yield) that was formed due to migration of a chloroacetyl group from O-7 to O-9. The structure of diol **7** was established by NMR spectroscopy, high-resolution mass spectrometry and X-ray diffraction analysis (see the Experimental section and Supporting Information File 1 for the details). Diol **7** was converted into glycosyl donor **2** by *O*-trifluoroacetylation with trifluoroacetic anhydride and sodium trifluoroacetate under previously developed [36,55] conditions.

### Supramer analysis

As we know that the concentrations of reactants can affect the outcome of glycosylation [31,33–34,37,39,43–44,46,56–57], one must choose a concentration at which to perform the test glycosylation. This can be done either by analogy with similar reactions or by utilizing some rationalization, the second approach is more attractive.

For the selection of concentrations, we used the supramer analysis [33–35,37,39,46,57–58] of solutions, which is an integral part of an approach that discusses the structure of a reaction solution in terms of the supramer hypothesis [35]. According to the supramer approach in many cases it is supramolecular aggregates (supramers) rather than single molecules of reacting substances that are the real reacting species [39]. This line of reasoning allowed us to explain, predict, discover a number of unusual phenomena and develop highly efficient and stereoselective glycosylation reactions with sialyl donors that lead to formation of Neu-α(2-3)-Gal [33–34,37] and Neu-α(2-6)-Gal [32,34,36] glycosidic linkages found in oligosaccharides [59] of biological and medical significance.

The supramer approach implies that changes in solute concentration can cause the supramers to rearrange and thus affect the structure of the solution in a discontinuous manner [33–35,37,39,46,56–58,60]. These abrupt changes (discontinuities) in the solution structure can be revealed by supramer analysis, which relies on the examination of the plots of numerical data that are related to the reaction solution such as the specific optical rotation [31,33,37,46,57,60–63], intensity of scattered light [33,37,57–58,60,62,64] or intensity of IR bands [31] against concentration for the presence of discontinuities. The concentrations corresponding to the discontinuities found are taken as critical [57,62] concentrations that separate the concentration ranges, where supramers of similar structures hence chemical properties exist, from other concentration ranges, where supramers that are organized differently and have modified chemical properties (reactivity, selectivity) are present [35]. The supramer analysis is able to distinguish solutions (of the same solute but with different concentrations) that have distinct solution structures [35,39].

In the context of this study, the primary objective of using supramer analysis is to rationally select concentrations for glycosylation [37] (see also the discussion below). In other words, the rational selection of concentrations for performing glycosylation reactions takes into consideration changes in solution structure with concentration.

**Figure 1:**
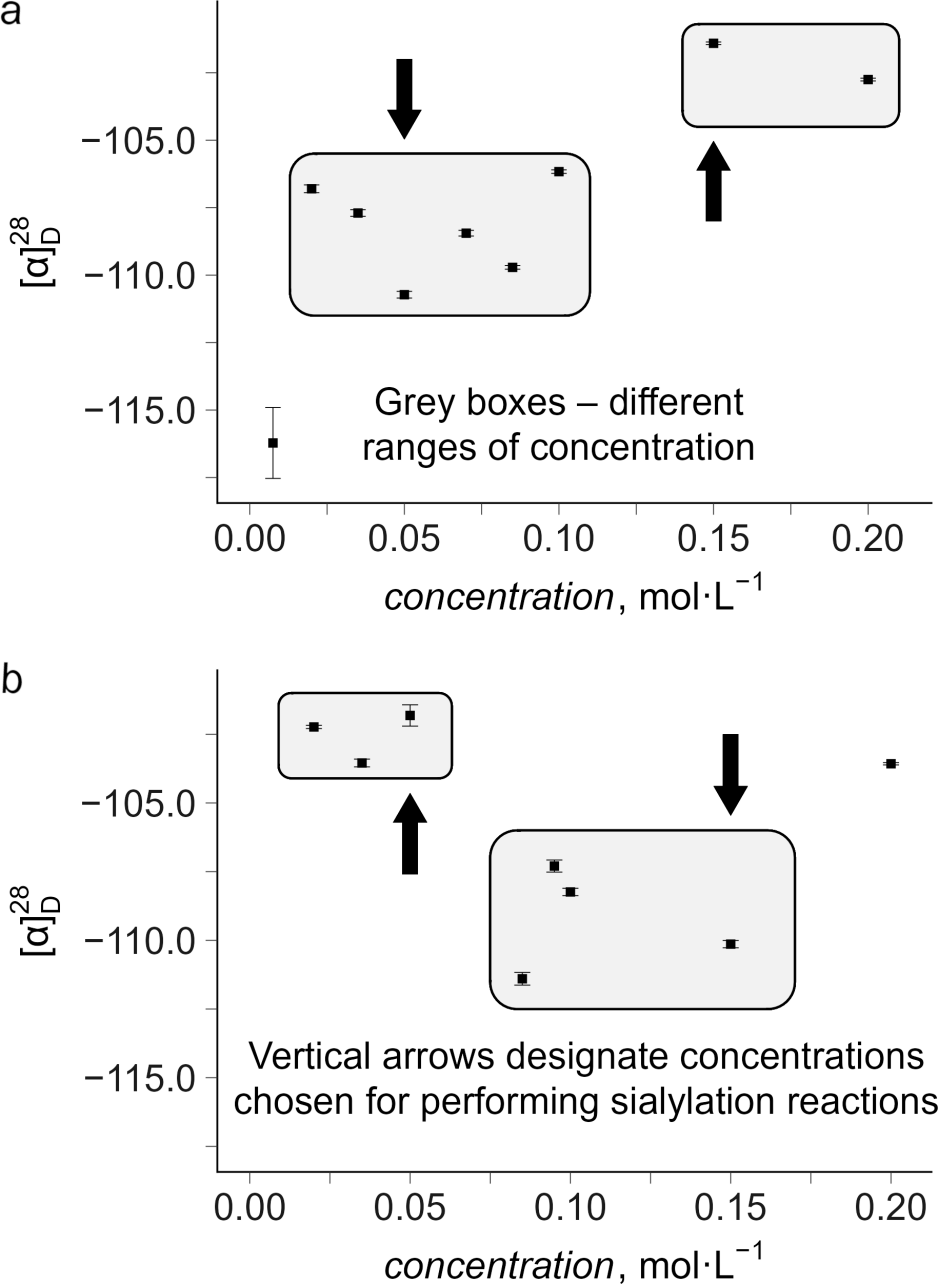
Concentration dependence of the specific optical rotation ([α]_D_^28^ / deg·dm^−1^·cm^3^·g^−1^) of solutions of sialyl donor **1** (a) and sialyl donor **2** (b) in MeCN at 28 °C. Each point represents an average of ten measurements (relative error <1% unless specified otherwise, see the error bars; the error bar is on the order of the symbol size if not visible). The standard deviations were calculated by using the Student’s distribution (95% probability). The grey boxes in the figure are drawn to guide the eye and designate different ranges of concentrations. Vertical arrows designate concentrations (0.05 and 0.15 mol·L^−1^) chosen for performing sialylation reactions. See also Table 1.

Accordingly, before carrying out the glycosylation experiments, we investigated, similarly to the previous studies [31,33–34,37,46], solutions of sialyl donors **1** and **2** in the reaction solvent (MeCN) by polarimetry, which is known [31,33–35,37,46,57,60–63] to be highly sensitive to changes in the structure of solutions. Analysis of the concentration dependence of the specific optical rotation (SR, [α]_D_) of solutions of sialyl donor **1** in MeCN revealed a considerable scatter of SR values at different concentrations. For this reason, the classical version of the supramer analysis (vide supra) cannot be directly used for revealing the critical concentrations and the corresponding concentration ranges featured by different supramers. However, a more careful examination of the data clearly suggests the presence of two concentration ranges, where the differences in SR values are statistically significant (see the grey boxes in Figure 1a): the high concentration range (*c* = 0.15–0.20 mol·L^−1^), where SR values are close to each other ([α]_D_^28^ = −102.1 ± 0.9 deg·dm^−1^·cm^3^·g^−1^) and the low concentration range (*c* = 0.02–0.10 mol·L^−1^), where the SR values are noticeably different from those in the high concentration range ([α]_D_^28^ = −108.3 ± 1.7 deg·dm^−1^·cm^3^·g^−1^). Similarly, two ranges of concentrations exist in solutions of sialyl donor **2** in MeCN (see the grey boxes in Figure 1b): the high concentration range (*c* = 0.085–0.15 mol·L^−1^), where [α]_D_^28^ = −109.3 ± 1.8 deg·dm^−1^·cm^3^·g^−1^) and the low concentration range (*c* = 0.02–0.05 mol·L^−1^), where [α]_D_^28^ = −102.5 ± 0.9 deg·dm^−1^·cm^3^·g^−1^.

According to the supramer approach [35,37,46,57,60], similar SR “values at different concentrations (for the same compound dissolved in the same solvent) suggest similar structures of supramers present at these concentrations, hence similar chemical properties of the solute” as described previously [37]. Conversely, the observed differences in SR values of solutions belonging to different concentration ranges are associated with changes in solution structure (see [33,37,39,46,57,60,62–63] for the details). This observation suggests that different types of supramers of each sialyl donor could be present in the high and low concentration ranges shown in Figure 1. Based on previous experience [33,37,46,57], we may expect different reactivity patterns of sialyl donors **1** and **2** in these concentration ranges.

We do understand that any model study of a structure of solution of pure glycosyl donor in a reaction solvent might not be as relevant when the reaction solution contains also glycosyl acceptor, promoter, suspended molecular sieves, etc., in addition to the glycosyl donor, and the presence of these components may also affect [31–33] solution structure and hence the glycosylation outcome. Although polarimetry data clearly suggest, in our opinion, the presence of different supramers in solutions of glycosyl donors **1** and **2** with different concentrations at ambient temperature (28 °C) (Figure 1, Table 1), the situation may be different at much lower temperatures (≤−40 °C), at which most sialylation reactions are performed. We do understand these limitations of the supramer analysis. Yet, the use of supramer analysis proved to be useful for selection of concentrations for performing chemical experiments and made possible the discovery of the previously unknown phenomenon [31,33–34,37,39,46,56–57] of bimodality of glycosylation (see [39] for more detailed discussion). At the current level of development, one may safely consider the supramer analysis at least as a pure heuristic tool for choosing experimental conditions.

**Table 1:** SR values and types of supramers in solutions of sialyl donors **1** and **2** at different concentrations.

Entry	Glycosyl donor	Concentration, mol·L^−1^	SR^a^	Supramer type

1	**1**	0.05	−110.7 ± 0.1	**1**-I
2	**1**	0.15	−101.4 ± 0.1	**1**-II
3	**2**	0.05	−101.8 ± 0.4	**2**-III
4	**2**	0.15	−110.1 ± 0.1	**2**-IV

^a^SR = [α]_D_^28^, deg·dm^−1^·cm^3^·g^−1^.

### Experimental design

After establishing the presence of the concentration ranges, in which different supramers of sialyl donors **1** or **2** are putatively present, we decided to perform comparative glycosylation experiments at two representative concentrations of the glycosyl donors (0.05 and 0.15 mol·L^−1^) that belong to these concentration ranges (see the vertical arrows in Figure 1 and data in Table 1). Although several concentrations from the low concentration range can be used for comparative glycosylation, we chose the concentration 0.05 mol L^−1^, which is the “regular” concentration commonly used for glycosylation by many research groups. For this reason, it makes sense to include this concentration in the experimental design to enable a direct comparison with data already published in the field. In addition, it is the highest concentration that belongs to the low concentration range found for compound **2** (Figure 1b). The concentration 0.15 mol·L^−1^ is the only concentration that belongs to the high concentration ranges for both compounds. Indeed, it is the lowest concentration that belongs to the high concentration range found for compound **1** (Figure 1a) and the highest concentration that belongs to the high concentration range found for compound **2** (Figure 1b).

Note the considerable differences in the SR values for solutions of sialyl donor **1** or sialyl donor **2** in MeCN at the chosen concentrations (compare entries 1 and 2 for sialyl donor **1** and entries 3 and 4 for sialyl donor **2** in Table 1). These differences in SR values suggest that supramers **1**-I and **1**-II of sialyl donor **1** and supramers **2**-III and **2**-IV of sialyl donor **2**, differing in their structures, could be present at these concentrations, respectively (Table 1). Since different supramers of the same compound may differ in their properties [35,39], we will in fact be comparing chemical properties of supramers **1**-I and **2**-III when performing glycosylation at the concentration of 0.05 mol·L^−1^, while at a concentration of 0.15 mol·L^−1^ chemical properties of supramers **1**-II and **2**-IV will be compared. Under this scenario, one might expect conflicting results of comparison of sialyl donors **1** and **2** performed at the selected concentrations (0.05 and 0.15 mol·L^−1^). Note that limitations of supramer analysis mentioned above apply here, too.

### Glycosylation experiments

In order to verify this prediction, we then performed two sets of comparative glycosylation experiments with equimolar amounts of sialyl donors **1** or **2** and glycosyl acceptor **3** (Scheme 1) under standard conditions (NIS, TfOH, MeCN, MS 3 Å, −40 °C) at the two chosen concentrations (0.05 and 0.15 mol·L^−1^). We intentionally took sialyl donors **1** or **2** and glycosyl acceptor **3** in equimolar quantities since “this experimental design allows easy monitoring the reaction course and correct estimation of time required for the reaction to complete. The use of excess of a sialyl donor is quite a common practice; in such cases, higher yields of glycosylation products are usually obtained in line with general consensus that the competing elimination from a sialyl donor is the main reason for diminished yields in sialylation” as described previously [37].

Due to possible lability of *O*-trifluoroacetyl groups, the crude product was treated with MeONa in MeOH in order to remove all *O*-acyl groups and then with Ac_2_O in Py to install *O*-acetyl groups. After this procedure the initially formed disaccharides with various *O*-protective groups were transformed to the same known disaccharide **4** [36]. This procedure significantly simplifies the analysis of the reaction results and purification of the product.

For the correct determination of the stereoselectivity of sialylation, it is important to analyze anomeric ratio values (α/β) for the disaccharide fraction separated by size-exclusion chromatography since the retention values of different disaccharide anomers on silica gel may be surprisingly large and a minor isomer may be lost during purification by silica gel chromatography. On the other hand, NMR analysis of the crude reaction mixtures may be misleading due to possible overlap of the signals of H-3_eq_ belonging both to disaccharide and monosaccharide derivatives sometimes present in such glycosylation mixtures.

**Figure 2:**
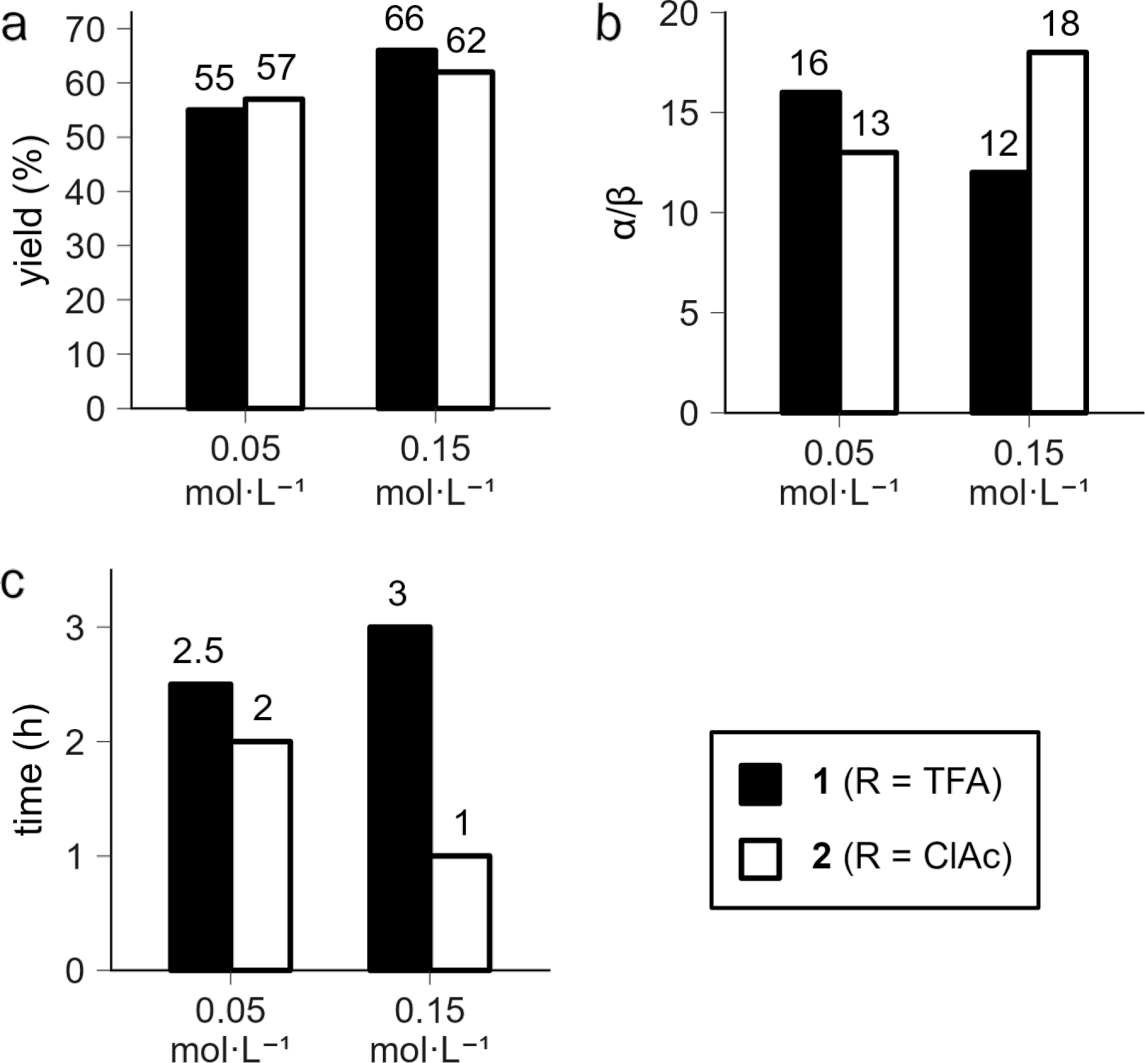
Comparison of the outcome of the sialylation of glycosyl acceptor **3** with sialyl donors **1** or **2** performed at different concentrations: yield of disaccharide **4** [36] (a), stereoselectivity (α/β) (b), and reaction time (c). See Scheme 1 for the structures of the compounds. The reaction was quenched after complete consumption of glycosyl donors (TLC control). The crude product was treated with MeONa in MeOH in order to remove all *O*-acyl groups and then with Ac_2_O in Py to install *O*-acetyl groups. The disaccharide fraction was isolated by gel permeation chromatography on Bio-Beads S-X3 (toluene) and analyzed by ^1^H NMR spectroscopy to give the anomeric ratio (α/β). Individual anomers of disaccharides were then separated by silica gel chromatography to give the yield.

At low concentration (0.05 mol·L^−1^) differences between the reactions involving sialyl donors **1** and **2** are small (see Figure 2 and Table S1 in Supporting Information File 1). Noteworthy is a slightly higher stereoselectivity achieved when sialyl donor **1** with a trifluoroacetyl group at O-9 was used (α:β = 16:1 for **1** versus α:β = 13:1 for **2**). One could speculate that a more nucleophilic carbonyl oxygen of the chloroacetyl group at O-9 in sialyl donor **2** might participate in a stabilization of the intermediate glycosyl cation from the α-side (as we discussed earlier [52–53]) diminishing the α/β ratio.

Conversely, at high concentration (0.15 mol·L^−1^) the differences between the reactions involving sialyl donors **1** and **2** are more profound (see Figure 2 and Table S1 in Supporting Information File 1). When sialyl donor **2** with a chloroacetyl group at O-9 was used the reaction proceeded three times faster (1 h for **2** versus 3 h for **1**) and the stereoselectivity was considerably higher (α:β = 18:1 for **2** versus α:β = 12:1 for **1**). The diminished reactivity of sialyl donor **1** at a higher concentration is surprising in view of the “law of mass action”.

At low concentration (0.05 mol·L^−1^) sialyl donor **1** with a trifluoroacetyl group at O-9 was more α-selective (23% difference) while at high concentration (0.15 mol·L^−1^) sialyl donor **2** with a chloroacetyl group at O-9 was more α-selective (50% difference) and the relative difference in stereoselectivity increased at high concentration (0.15 mol·L^−1^). Importantly, clear reasons for the observed increase in the reactivity and selectivity for sialyl donor **2** with a chloroacetyl group at O-9 at high concentration (0.15 mol·L^−1^) are missing.

All this suggests that in the particular case of sialyl donors **1** and **2** studied it is not possible to conclude unambiguously which one of them is more reactive and which one of them provides higher stereoselectivity in the sialylation of the same glycosyl acceptor **3**. Thus, it is not possible to reveal a possible influence of remote acyl protective groups at O-9 on the sialylation outcome, hence experimentally confirm or disprove their putative participation in stabilization [52–53] of the glycosyl cation. More importantly, in our opinion, this result indicates the existence of unexpected difficulties in the determination of relative reactivities of glycosyl donors (vide infra).

## Discussion

It is generally believed that the molecular structure and the reaction mechanism are the keys to understanding chemical reactivity and selectivity [65–67]. In the area of carbohydrate chemistry, a lot of efforts are devoted to finding relationships between the fine details of molecular structures of both glycosylation partners (glycosyl donor [22–23,68–71] and glycosyl acceptor [26,72]) and their reactivity and stereoselectivity of glycosylation reactions in which they participate. Importantly, the comparative studies [69–72] are at the core of this approach. However, reactivity and stereoselectivity cannot be considered as the features of molecular structures of the glycosyl donor and acceptor since other factors may become more important. For example, it was reported that “glycosylations involving two specific partners can be tuned to produce either 11:1 selectivity of one stereoisomer or 9:1 of the other by merely changing the reaction conditions” [73–74]. In our opinion, these “environmental variables” [73] (including concentration of reagents) can influence structure and/or composition of supramers of reagents formed in reaction solutions. These changes may result in a modulation of the conformation or the presentation [39] (i.e., microenvironment) of glycosyl donor molecules, which are incorporated in supramers, hence their chemical properties [35], thus making possible a shift of a fragile and not well-understood borderline between different reaction pathways at the S_N_1–S_N_2 interface [22,24–25], which could modulate the stereoselectivity of glycosylation and the observed reactivity pattern.

After the dramatic dependence of the sialylation outcome on concentration has been discovered [33], it became common (especially in the thoroughly performed studies) to indicate the concentration used when reporting the results of glycosylation [39,43–44,46]. For this reason, it seems quite natural to compare different glycosyl donors at identical concentrations. It is this approach that is used for the determination of relative reactivities of glycosyl donors (RRV values) [69–71] and glycosyl acceptors (Aka values) [72]. Surprisingly, as the results obtained in this study suggest, such comparison of results of glycosylation experiments at identical concentrations is not enough to get reliable data on the relative reactivity and stereoselectivity of glycosyl donors even if the structural differences are small (as in the case of silayl donors **1** and **2**).

In this study, each glycosylation experiment was performed only once, which is currently a common practice in this area of research. Since the assessment of errors is not possible in such a case, it is not possible to estimate correctly whether the differences in reaction outcome are statistically significant. Importantly, a similar conclusion is applicable to the majority of already published data on comparative glycosylations.

Our results seem to question the universal validity of the comparative data already published. Indeed, reactions performed with identical pairs of glycosyl donor and glycosyl acceptor at another concentration (which corresponds to a different set of “environmental variables” [73]) may well give completely different results, which would prove an alternative theory about how a specific group (be it a (dis)arming or stereodirecting group) exerts its influence on the outcome of glycosylation.

This puzzling conclusion deserves a comment. The issue of inconsistent results of comparison of glycosyl donors performed at different concentrations is completely resolved if one recalls the basics of the supramer hypothesis which postulates that it is the supramers that are the real reactive species in solutions. Since the comparison of sialyl donors **1** and **2** was performed at two concentrations where different types of supramers, which incorporate molecules of the corresponding glycosyl donors, are expected to exist (see Table 1), it is quite natural to expect for these differently arranged supramers to have different chemical properties in glycosylation experiments performed at these two concentrations [35]. Indeed, changes in “packing“ mode of sialyl donor molecules incorporated in the corresponding supramers or their “presentation” [38–39] on the surface of the supramers may induce conformational changes in the pyranose ring [24,47–48,52–53] or the side chain [24,48,75–80], which would modulate the proximity of O-9 to the anomeric center C-2, hence the influence of the nature of a substituent at O-9 on the sialylation outcome. In actual glycosylation experiments (Scheme 1, Figure 2), in our opinion, we compared the behavior of the supramers formed at particular concentrations (supramers **1**-I and **2**-III at concentration 0.05 mol·L^−1^ and supramers **1**-II and **2**-IV at concentration 0.15 mol·L^−1^, see Table 1) rather than that of the parent molecules of sialyl donors **1** and **2**.

Then it is no longer surprising why at low concentration (0.05 mol·L^−1^) the sialyl donor **1** was 23% more stereoselective while at high concentration (0.15 mol·L^−1^) sialyl donor **2** was 50% more stereoselective and the relative difference in stereoselectivity between sialyl donors **1** and **2** increased at high concentration (0.15 mol·L^−1^) (see Figure 2b). We must stress that any alternative explanation of the differences in stereoselectivity should explain why sialyl donor **2** with a chloroacetyl group at O-9 became 38% more stereoselective while sialyl donor **1** with a trifluoroacetyl group at O-9 became 33% less stereoselective at high concentration (0.15 mol·L^−1^) as compared to the results at low concentration (0.05 mol·L^−1^). To the best of our knowledge, traditional mechanistic reasons for the opposite influence of the increase in concentration on stereoselectivity achieved with different glycosyl donors are currently missing. A putative cross-over from one reaction pathway to another one within the S_N_1–S_N_2 continuum [22,24–25] would require a sensible explanation why this happens differently for the two glycosyl donors under study.

One should also bear in mind that here we (as most other researchers do in similar situations) are talking about the starting concentrations of reactants only. During the course of the glycosylation reaction the concentrations of reactants (as well as concentrations of reaction products) would inevitably change. This could lead to modifications of the structure of the corresponding supramers of reactants, hence their properties. Indeed, we have already addressed this issue and found that the stereoselectivity of the glycosylation of alcohol **3** by a related *N*,*O*-acetylsialyl thioglycoside may depend on the reaction time (generally, decrease in time) [32,34]. In the case of glycosyl donors **1** and **2**, these changes of concentrations could shift the reaction solution structure to another range of concentration (Figure 1), featured by the presence of different supramers (Table 1), and affect the reactivity pattern. However, analysis of the results obtained (Figure 1b and Table S1 in Supporting Information File 1) clearly suggests that, again, there is no universal correlation between reaction time and stereoselectivity. Although at high concentration (0.15 mol·L^−1^) an increase in the reaction time resulted in a decrease in stereoselectivity (analogously to the previous results [32,34]), at low concentration (0.05 mol·L^−1^) the opposite trend was observed (here, we, for the moment, ignore structural differences between glycosyl donors **1** and **2**; similar conclusions can be made if we compare results of glycosylations performed with glycosyl donors **1** or **2** separately). Again, a sensible explanation (without resorting to suparmer approach) of different temporal dependences of stereoselectivity for the two different glycosyl donors and different concentrations would be required.

Although in this particular case no additional physicochemical studies were attempted to support our rationalization, which is based on polarimetry data only (polarimetry is currently “the method of choice for supramer analysis of solutions” due to its exceptional sensitivity to changes in solution structure [60–63]), previous experience suggests that the unexpected diminished reactivity of sialyl donor **1** observed at high concentration (0.15 mol·L^−1^) might be related to the formation of more tight and less reactive supramers (similar to those discovered recently [37–39,58]), in which the molecules of sialyl donor **1** located in the supramer core are not easily accessible for other reagents and therefore cannot efficiently participate in the glycosylation reaction. We must stress that any alternative explanation of the differences in reactivities of the studied glycosyl donors should also explain why these differences can be detected only at some selected concentrations.

More pronounced differences in reactivities between sialyl donors **1** and **2** and higher α-selectivity achieved with sialyl donor **2** at high concentration (0.15 mol·L^−1^) (see Figure 2) may well indicate that upon an increase in concentration a shift from the S_N_1-like mechanism to a more S_N_2-like mechanism occurs only (or mainly) for sialyl donor **2**. Although this hypothesis can explain why the glycosylation with sialyl donor **2** exhibits substantial concentration dependence, it does not allow one even to guess why such S_N_1-to-S_N_2 cross-over did not occur for sialyl donor **1**.

In order to exclude misunderstanding, some comments are required concerning the use of supramer analysis. We use the supramer analysis mostly as a heuristic tool that could suggest a set of concentrations at which to perform actual glycosylation reactions by an educated guess rather than by trial and error. And indeed, in this study, such a strategy again [33–35,37,46,57] led to rather strange and unexpected results when comparing two glycosylation reactions performed at two different concentrations that were chosen based on the results of supramer analysis. Importantly, it is the supramer analysis that allowed us to reveal this new knowledge. Sialyl donor **1** has earlier been used in sialylation only at a single “regular” concentration (50 mmol·L^−1^) [36] and its solutions have never been studied by supramer analysis. Only in this study we performed a comparison of the reaction outcomes at different concentrations. Sialyl donor **2** was not studied previously.

It is not improbable that a choice of an alternative set of concentrations (e.g., selected randomly) would provide different results, which could well seem to be trivial. This conclusion of the usefulness of the supramer analysis (and the supramer approach in general) is valid irrespective of the precise reasons behind the behavior of the studied glycosyl donors. We have to emphasize that providing conclusive evidence of the real existence of supramers of reagents in the reaction mixtures and their influence on the reaction outcome is far beyond the scope of this study.

## Conclusion

In conclusion, an attempted comparison of chemical properties of two sialyl donors (**1** and **2**) at different concentrations, which were chosen basing on results of the supramer analysis, led to mutually excluding conclusions concerning their relative reactivity and selectivity. This prevented us from revealing a possible influence of remote acyl protective groups at O-9 on the sialylation outcome and their role in stabilization of glycosyl cation in a conformation with all-axial substituents in the pyranose ring. According to the results of the supramer analysis of reaction solutions, this issue might be related to the formation of supramers of glycosyl donors differing in structure hence chemical properties. Even more importantly, these results seem to imply that a comparison of chemical properties of different glycosyl donors may not be as simple and straightforward as it is usually considered. Similar conclusions might be applicable to other systems, too. These results provide a fresh insight into the problems of reactivity of chemical compounds and the selectivity of the reactions in which they participate.

## Experimental

### General methods

All methods and procedures followed those described in our previous publications [33,36–37]. The reactions were performed with the use of commercial reagents. Solvents for reactions were distilled and purified before the use according to the standard procedures. MeCN for glycosylation reactions was distilled under argon over P_2_O_5_ and then over CaH_2_ and stored over molecular sieves (MS) 3 Å. Powdered MS 3 Å (Fluka) were activated before the reactions by heating at ≈220 °C in high vacuum for 6 h. Column chromatography was performed on silica gel 60 (40–63 μm, Merck). Gel permeation chromatography was performed in toluene on a column (400 × 20 mm) packed with Bio-Beads S-X3 gel (200–400 mesh, Bio-Rad) using a differential refractive index detector (Knauer). TLC was carried out on Silica Gel 60 F_254_ plates (Merck), spots were visualized under UV light and by heating plates after immersion in a 1:10 (v/v) mixture of 85% aq H_3_PO_4_ and 95% aq EtOH. ^1^H, ^13^C, and ^19^F NMR spectra of solutions in CDCl_3_ and acetone-*d*_6_ were recorded on a Bruker AVANCE-600 instrument at 600 MHz for ^1^H and 151 MHz for ^13^C or on a Bruker AM-300 instrument at 300 MHz for ^1^H, 75 MHz for ^13^C, and 282 MHz for ^19^F NMR. The ^1^H chemical shifts are given relative to the signal of the residual CHCl_3_ (δ_H_ 7.27) or acetone-*d*_5_ (δ_H_ 2.05), the ^13^C chemical shifts were measured relative to the signal of CDCl_3_ (δ_C_ 77.0) or acetone-*d*_6_ (δ_C_ 29.92). The ^19^F chemical shifts are given relative to the external signal of CFCl_3_ (δ_F_ 0.0). Assignments of the signals in the NMR spectra were performed using 2D-spectroscopy (COSY, HSQC, HMBC) and DEPT-135 experiments. For the copies of NMR spectra for all new compounds see Supporting Information File 1. High resolution mass spectra (electrospray ionization, HRESIMS) were measured in a positive mode on a Bruker micrOTOF II mass spectrometer for 2·10^−5^ M solutions in MeCN as described previously [36].

### Experimental procedure for optical rotation measurements

The procedure for optical rotation measurements followed that described in our previous publications [37,46]. Optical rotation values were measured with a PU-07 automatic digital polarimeter (Russia) at 28 °C in a jacketed glass cell (10 cm length). Special precautions were made to ensure the stability of the instrument and the temperature within the measuring compartment of the instrument and the cell, which was maintained with an accuracy of ±0.2 °C. After the instrument was warmed up for at least 1 h (as experience suggests, after this period the temperature within the instrument remains stable for at least 8–10 h of continuous work) the instrument readings were verified against the quartz standards (α = +21.267 and −21.248) as described previously [37]. After initial thermal stabilization of the sample (placed in a jacketed cell connected to a circulating water thermostat) within the instrument (10 min), a series of 10 successive measurements of the sample was made within a 2–3 min period followed by another measurement of the quartz standards (to monitor the instrument stability). Each measurement at a particular concentration was repeated 10 times, then averaged and plotted against concentration. The standard deviations were calculated by using the Student’s distribution (95% probability) and did not exceed 1% for either observed (α_D_) or specific ([α]_D_) rotation values. The concentrations (*c*) of the solutions used for calculating the specific optical rotation (SR) are given in traditional polarimetric units (g/100 mL) unless otherwise explicitly stated as described previously [46].

### Synthesis and characterization

#### Methyl (phenyl 4,9-bis-*O*-chloroacetyl-3,5-dideoxy-2-thio-5-trifluoroacetamido-7,8-bis-*O*-trifluoroacetyl-ᴅ-glycero-β-ᴅ-galacto-nonulopyranosid)onate (**2**)

To the solution of diol **7** (124.9 mg, 0.2 mmol, dried in vacuo at 0.1 Torr for 1 h) in (CF_3_CO)_2_O (4 mL), sodium trifluoroacetate (25 mg, freshly dried at 80 °C in vacuo (0.1 Torr, 1 h)) was added. The mixture was stirred at room temperature (≈20 °C) until complete consumption of the starting material (TLC monitoring, *R*_f_ = 0.10 (**7**), *R*_f_ = 0.57 (**2**), benzene/EtOAc 9:1). The reaction mixture was concentrated under reduced pressure, the residue was triturated with anhydrous benzene (5 mL), and the extract was filtered through a PTFE microfilter (0.45 μm, 13 mm diameter, Iso-Disk, Supelco). The filtrate was concentrated under reduced pressure and the residue was dried in vacuo to give **2** as a colorless solid (118.8 mg, 73%). [α]_D_^19^ −87.4 (*c* 4.3, CHCl_3_); *R*_f_ 0.57 (benzene/EtOAc 9:1); ^1^H NMR (300 MHz, CDCl_3_, δ, ppm, *J*, Hz) 2.25 (dd, *J*_3a,3e_ = 13.9, *J*_3a,4_ = 11.7, 1H, H-3a), 2.82 (dd, *J*_3e,3a_ = 13.9, *J*_3e,4_ = 4.8, 1H, H-3e), 3.71 (s, 3H, OMe), 3.96 (ABq, ΔδAB = 0.03, *J*_AB_ = 15.0, 2H, CH_2_Cl), 4.09 (ABq, ΔδAB = 0.02, *J*_AB_ = 15.4, 2H, CH_2_Cl), 4.12 (dd, *J*_9a,9b_ = 12.5, *J*_9a,8_ = 8.1, 1H, H-9a), 4.18 (ddd, *J*_5,6_ = 10.6, *J*_5,4_ = 10.3, *J*_5,NH_ = 9.9, 1H, H-5), 4.64 (dd, *J*_9b,9a_ = 12.5, *J*_9b,8_ = 2.6, 1H, H-9b), 4.89 (dd, *J*_6,5_ = 10.6, *J*_6,7_ = 2.2, 1H, H-6), 5.11 (ddd, *J*_8,9a_ = 8.1, *J*_8,9b_ = 2.6, *J*_8,7_ = 2.2, 1H, H-8), 5.55 (dd, *J*_7,6_ = 2.2, *J*_7,8_ = 2.2, 1H, H-7), 5.81 (ddd, *J*_4,3a_ = 11.7, *J*_4,5_ = 10.3, *J*_4,3e_ = 4.8, 1H, H-4), 7.27 (d, *J*_NH,5_ = 9.9, 1H, NH), 7.33–7.49 (m, 5H, Ph); ^13^C NMR (75 MHz, CDCl_3_, δ, ppm, *J*, Hz) 37.0 (C-3), 40.1 (CH_2_Cl), 40.3 (CH_2_Cl), 49.8 (C-5), 53.1 (OMe), 62.5 (C-9), 70.1 (C-4), 71.5, 71.8 (C-6, C-7), 75.5 (C-8), 88.2 (C-2), 114.1 (q, *J*_C,F_ = 285, CF_3_), 114.2 (q, *J*_C,F_ = 285, CF_3_), 115.2 (q, *J*_C,F_ = 288, CF_3_), 127.6, 129.6, 130.6, 136.0 (Ph), 156.4 (q, *J*_C,F_ = 45, COCF_3_), 156.9 (q, *J*_C,F_ = 44, COCF_3_), 158.0 (q, *J*_C,F_ = 39, COCF_3_), 166.2, 167.5, 168.4 (COCH_2_Cl, CO_2_Me); ^19^F NMR (282 MHz, CDCl_3_, δ, ppm) −76.8 (NHCOCF_3_), −75.7, −75.2 (OCOCF_3_); HRESIMS (*m*/*z*): [M + NH_4_]^+^ calcd for C_26_H_26_Cl_2_F_9_N_2_O_12_S, 831.0434; found, 831.0421.

#### Methyl (phenyl 4,7-bis-*O*-chloroacetyl-3,5-dideoxy-8,9-*O*-isopropylidene-2-thio-5-trifluoroacetamido-ᴅ-glycero-β-ᴅ-galacto-nonulopyranosid)onate (**6**)

In a manner similar to [36], to the solution of diol **5** [36] (900 mg, 1.77 mmol) in anhydrous CH_2_Cl_2_ (8.0 mL), 2,4,6-collidine (1.4 mL, 10.6 mmol) was added. The solution was cooled to 0 °C (ice–water bath), and a solution of chloroacetic anhydride (906 mg, 5.30 mmol) in anhydrous CH_2_Cl_2_ (11.5 mL) was added dropwise while stirring. The reaction mixture was stirred at 0 °C for 70 min until complete consumption of the starting material (TLC monitoring, *R*_f_ = 0.10 (**5**), *R*_f_ = 0.49 (**6**), EtOAc/petroleum ether 35:65). Saturated aqueous NaHCO_3_ (5 mL) was added, and the mixture was well shaken and then allowed to warm to ≈20 °C, diluted with CH_2_Cl_2_ (50 mL), washed with water (100 mL), 1 M aqueous NaHSO_4_ (100 mL), and saturated aqueous NaHCO_3_ (100 mL). An additional extraction with CH_2_Cl_2_ (2 × 50 mL) was performed from each aqueous layer. The combined organic extracts were filtered through a cotton wool plug, concentrated under reduced pressure, and dried in vacuo as described previously [36]. The residue was purified by silica gel chromatography (column volume 140 mL, eluent EtOAc/petroleum ether 25:75) to give **6** as a colorless foam (1.06 g, 90%). [α]_D_^23^ −105.5 (*c* 1.0, CHCl_3_); *R*_f_ 0.58 (benzene/CH_2_Cl_2_/acetone 2:2:0.8); ^1^H NMR (300 MHz, CDCl_3_, δ, ppm, *J*, Hz) 1.31 (s, 3H, Me), 1.34 (s, 3H, Me), 2.21 (dd, *J*_3a,3e_ = 13.8, *J*_3a,4_ = 11.4, 1H, H-3a), 2.86 (dd, *J*_3e,3a_ = 13.8, *J*_3e,4_ = 4.8, 1H, H-3e), 3.60 (s, 3H, OMe), 3.66 (dd, *J*_9a,9b_ = 9.2, *J*_9a,8_ = 6.6, 1H, H-9a), 3.85 (dd, *J*_9b,9a_ = 9.2, *J*_9b,8_ = 6.6, 1H, H-9b), 4.02 (ddd, *J*_8,9a_ = 6.6, *J*_8,9b_ = 6.6, *J*_8,7_ = 4.4 Hz, 1H, H-8), 4.05 (s, 2H, CH_2_Cl), 4.08 (ddd, *J*_5,4_ = 10.6, *J*_5,NH_ = 10.3, *J*_5,6_ = 10.3, 1H, H-5), 4.13 (ABq, ΔδAB = 0.03, *J*_AB_ = 14.7, 2H, CH_2_Cl), 4.76 (dd, *J*_6,5_ = 10.3, *J*_6,7_ = 2.2, 1H, H-6), 5.45 (dd, *J*_7,8_ = 4.4, *J*_7,6_ = 2.2, 1H, H-7), 5.77 (ddd, *J*_4,3a_ = 11.4, *J*_4,5_ = 10.6, *J*_4,3e_ = 4.8, 1H, H-4), 6.99 (d, *J*_NH,5_ = 10.3, 1H, NH), 7.33–7.50 (m, 5H, Ph); ^13^C NMR (75 MHz, CDCl_3_, δ, ppm, *J*, Hz) 25.0 (Me), 26.2 (Me), 37.7 (C-3), 40.3 (CH_2_Cl), 40.6 (CH_2_Cl), 50.2 (C-5), 52.7 (OMe), 65.2 (C-9), 70.5 (C-4), 70.7 (C-7), 71.2 (C-6), 74.7 (C-8), 88.7 (C-2), 108.6 (*C*Me_2_), 115.3 (q, *J*_C,F_ = 288, CF_3_), 128.5, 129.0, 130.2, 136.1 (Ph), 157.8 (q, *J*_C,F_ = 38, *C*OCF_3_), 166.7 (*C*OCH_2_Cl), 167.6 (*C*O_2_Me), 168.0 (*C*OCH_2_Cl); ^19^F NMR (282 MHz, CDCl_3_, δ, ppm) −76.7; HRESIMS (*m*/*z*): [M + Na]^+^ calcd for C_25_H_28_Cl_2_F_3_NNaO_10_S, 684.0655; found, 684.0649.

#### Methyl (phenyl 4,9-bis-*O*-chloroacetyl-3,5-dideoxy-2-thio-5-trifluoroacetamido-ᴅ-glycero-β-ᴅ-galacto-nonulopyranosid)onate (**7**)

To the solution of 8,9-*O*-isopropylidene derivative **6** (871 mg, 1.4 mmol) in CH_2_Cl_2_ (60 mL), 90% aq CF_3_CO_2_H (6 mL, freshly prepared) was added at 0 °C (ice–water bath). The reaction mixture was stirred at 0 °C for 25 min. The mixture was allowed to warm to ≈20 °C, toluene (20 mL) was added, and then concentrated. The residue was purified by silica gel column chromatography (column volume 140 mL, elution with gradient CH_2_Cl_2_ → acetone/CH_2_Cl_2_ 20:80). The crude product was dissolved in acetone (3 mL) and *t*-BuOMe (3 mL), then petroleum ether was slowly added until crystallization commenced. The precipitate formed was filtered off and washed with *t*-BuOMe/petroleum ether 1:1 (v/v) mixture to give **7** as white crystals (576 mg, 70%). [α]_D_^27^ −116.0 (*c* 2.9, acetone); *R*_f_ 0.21 (toluene/acetone 5:1); ^1^H NMR (600 MHz, acetone-*d*_6_, δ, ppm, *J*, Hz) 2.20 (dd, *J*_3a,3e_ = 13.6, *J*_3a,4_ = 11.9, 1H, H-3a), 2.83 (dd, *J*_3e,3a_ = 13.6, *J*_3e,4_ = 4.8, 1H, H-3e), 3.48 (s, 3H, OMe), 3.72 (ddd, *J*_7,OH_ = 9.1, *J*_7,8_ = 8.6, *J*_7,6_ = 1.0, 1H, H-7), 4.02 (dddd, *J*_8,7_ = 8.6, *J*_8,9a_ = 6.2, *J*_8,OH_ = 6.2, *J*_8,9b_ = 2.4, 1H, H-8), 4.07 (d, *J*_OH,7_ = 9.1, 1H, 7-OH), 4.25 (ABq, Δ*δ*AB = 0.06, *J*_AB_ = 14.3, 2H, CH_2_Cl), 4.26 (s, 2H, CH_2_Cl), 4.30 (d, *J*_OH,8_ = 6.2, 1H, 8-OH), 4.32 (dd, *J*_9a,9b_ = 11.4, *J*_9a,8_ = 6.2, 1H, H-9a), 4.45 (ddd, *J*_5,4_ = 10.5, *J*_5,6_ = 10.5, *J*_5,NH_ = 8.1, 1H, H-5), 4.51 (dd, *J*_9b,9a_ = 11.4, *J*_9b,8_ = 2.4, 1H, H-9b), 4.90 (dd, *J*_6,5_ = 10.5, *J*_6,7_ = 1.0, 1H, H-6), 5.60 (ddd, *J*_4,3a_ = 11.9, *J*_4,5_ = 10.5, *J*_4,3e_ = 4.8, 1H, H-4), 7.36–7.38 (m, 2H, *m*-Ph), 7.41–7.42 (m, 1H, *p*-Ph), 7.61–7.63 (m, 2H, *o*-Ph), 8.74 (d, *J*_NH,5_ = 8.1 Hz, 1H, NH); ^13^C NMR (151 MHz, acetone-*d*_6_, δ, ppm, *J*, Hz) 38.2 (C-3), 41.5 (CH_2_Cl), 41.8 (CH_2_Cl), 50.9 (C-5), 52.7 (OMe), 69.0 (C-9), 69.4 (C-8), 70.2 (C-7), 71.8 (C-4), 72.3 (C-6), 90.3 (C-2), 117.0 (q, *J*_C,F_ = 288, CF_3_), 129.8, 130.6, 137.3 (Ph), 158.4 (q, *J*_C,F_ = 38, *C*OCF_3_), 167.7 (*C*OCH_2_Cl), 168.1 (*C*OCH_2_Cl), 168.4 (*C*O_2_Me). Note: the values of the coupling constants (*J*_C,F_) were calculated using positions of the two central signals of the multiplet; two side lines of the multiplet are not visible due to low signal-to-noise ratio. ^19^F NMR (282 MHz, acetone-*d*_6_, δ, ppm) −77.1; HRESIMS (*m*/*z*): [M + Na]^+^ calcd for C_22_H_24_Cl_2_F_3_NNaO_10_S, 644.0342; found, 644.0349 . For the details of the single crystal X-ray analysis data for compound **7** (CCDC 1843708) see Supporting Information File 1 and Supporting Information File 2.

#### Typical glycosylation procedure

The glycosylation procedure followed that described in our previous publications [33,36–37]. A mixture of thioglycoside sialyl donor **1** [36] or **2** (1 equiv, 0.1 or 0.15 mmol) and alcohol **3** [54] (1 equiv) was dried in vacuo for 2 h, then anhydrous MeCN (2.0 mL for 0.1 mmol of sialyl donor and 1.0 mL for 0.15 mmol of sialyl donor) was added under argon. Freshly activated powdered MS 3 Å (Fluka; 100 mg per 1 mL of solvent) were added to the resulting solution. The suspension was stirred under argon at ≈20 °C for 1 h, then cooled to −40 °C (dry ice–MeCN bath). Solid NIS (1.5 equiv per 1 equiv of glycosyl donor) was added under argon followed by neat TfOH (2 μL, 0.02 mmol) to give a persistent iodine color as described previously [36]. The reaction mixture was stirred under argon at −40 °C until complete consumption of the starting thioglycoside (TLC monitoring, *R*_f_ 0.68 (**1**), *R*_f_ 0.57 (**2**), benzene/acetone 9:1), then diluted with CHCl_3_ (20 mL) and filtered through Celite pad. The solids were thoroughly washed with CHCl_3_ (100 mL) and the filtrate was successively washed with 20% aqueous Na_2_S_2_O_3_ (2 × 50 mL) and water (2 × 50 mL), filtered through a cotton wool plug and concentrated as described previously [33]. The residue was dissolved in anhydrous MeOH (3 mL per 0.1 mmol of sialyl donor) and MeONa (0.3 mL of 1 M solution in MeOH per 0.1 mmol of sialyl donor) followed by ethyl trifluoroacetate (0.1 mL per 0.1 mmol of sialyl donor) was added. The reaction mixture was kept at room temperature overnight, then quenched by addition of dry ice (solid CO_2_) and concentrated under reduced pressure. The residue was co-concentrated with toluene (3 mL), dried in vacuo, dissolved in anhydrous pyridine (3 mL per 0.1 mmol of sialyl donor), and acetic anhydride (3 mL per 0.1 mmol of sialyl donor) was added. The reaction mixture was kept at room temperature overnight, then quenched by addition of methanol (3 mL), and concentrated under reduced pressure. The residue was co-concentrated with toluene (3 mL), dissolved in CH_2_Cl_2_ (40 mL), washed with water (40 mL), the organic layer was filtered through a cotton wool plug and concentrated under reduced pressure. The residue was dried in vacuo, dissolved in toluene (2 mL) and separated by gel permeation chromatography on a Bio-Beads S-X3 column (toluene). The first eluted fraction contained disaccharides **4**, which was analyzed by NMR spectroscopy to give anomeric ratio values (α/β, see Figure 2 and Table S1 in Supporting Information File 1). For determination of the α/β ratio, integral intensities of signals of α-H-3eq and β-H-3eq of Neu5Ac residue of **4** were used (for relevant parts of the ^1^H NMR spectra see Figures S1–S4 in Supporting Information File 1). The disaccharide fraction was chromatographed on a silica gel 60 column (column volume 100 mL, eluted with gradient acetone/benzene 1:99 → 10:90) to give the known [36] pure α-linked isomer of disaccharide **4** as white foam (α-**4**, for yields see Figure 2 and Table S1 in Supporting Information File 1; all yields were calculated with respect to the glycosyl donor taken).

## Supporting Information

File 1 Copies of NMR spectra for all new compounds, single crystal X-ray analysis data for compound **7** (CCDC 1843708).

File 2 Deposed crystallographic information file (CCDC 1843708) and check file.

## Data Availability

All data that supports the findings of this study is available in the published article and/or the supporting information to this article.

## References

[1] Angata T, Varki A (2002). Chem Rev.

[2] Varki A (2007). Nature.

[3] Varki A (2008). Trends Mol Med.

[4] Schauer R (2009). Curr Opin Struct Biol.

[5] Chen X, Varki A (2010). ACS Chem Biol.

[6] Varki A, Gagneux P (2012). Ann N Y Acad Sci.

[7] Deng L, Chen X, Varki A (2013). Biopolymers.

[8] Schauer R, Kamerling J P (2018). Adv Carbohydr Chem Biochem.

[9] Sato C, Kitajima K (2019). Adv Carbohydr Chem Biochem.

[10] Blaum B S, Stehle T (2019). Adv Carbohydr Chem Biochem.

[11] Schnaar R L (2019). Adv Carbohydr Chem Biochem.

[12] Seeberger P H (2009). Nat Chem Biol.

[13] Solís D, Bovin N V, Davis A P, Jiménez-Barbero J, Romero A, Roy R, Smetana K, Gabius H-J (2015). Biochim Biophys Acta, Gen Subj.

[14] Stallforth P, Lepenies B, Adibekian A, Seeberger P H (2009). J Med Chem.

[15] Seeberger P H, Rademacher C (2014). Carbohydrates as drugs.

[16] Navuluri C, Crich D, Hung S-C, Zulueta M M L (2016). Stereocontrolled synthesis of sialosides. Glycochemical Synthesis: Strategies and Applications.

[17] Sun B (2016). Curr Org Chem.

[18] Lih Y-H, Wu C-Y, Bennett C S (2017). Chemical synthesis of sialosides. Selective Glycosylations: Synthetic Methods and Catalysts.

[19] De Meo C, Jones B T (2018). Adv Carbohydr Chem Biochem.

[20] De Meo C, Goeckner N, Barchi J J, Vidal S (2021). Synthesis of glycosides of sialic acid. Comprehensive Glycoscience.

[21] Vibhute A M, Komura N, Tanaka H-N, Imamura A, Ando H (2021). Chem Rec.

[22] Adero P O, Amarasekara H, Wen P, Bohé L, Crich D (2018). Chem Rev.

[23] Hettikankanamalage A A, Lassfolk R, Ekholm F S, Leino R, Crich D (2020). Chem Rev.

[24] Crich D (2021). J Am Chem Soc.

[25] Andreana P R, Crich D (2021). ACS Cent Sci.

[26] van der Vorm S, Hansen T, van Hengst J M A, Overkleeft H S, van der Marel G A, Codée J D C (2019). Chem Soc Rev.

[27] Ágoston K, Watt G M, Barchi J J, Vidal S (2021). Methods for O-glycoside synthesis. Comprehensive Glycoscience.

[28] Ishiwata A, Tanaka K, Ao J, Ding F, Ito Y (2022). Front Chem (Lausanne, Switz).

[29] Mukherjee M M, Ghosh R, Hanover J A (2022). Front Mol Biosci.

[30] Kononov L O, Malysheva N N, Kononova E G, Garkusha O G (2006). Russ Chem Bull.

[31] Kononov L O, Malysheva N N, Kononova E G, Orlova A V (2008). Eur J Org Chem.

[32] Kononov L O, Malysheva N N, Orlova A V (2009). Eur J Org Chem.

[33] Kononov L O, Malysheva N N, Orlova A V, Zinin A I, Laptinskaya T V, Kononova E G, Kolotyrkina N G (2012). Eur J Org Chem.

[34] Kononov L O, Taylor J C (2013). Modulation of stereoselectivity of glycosylation: a supramer approach. Advances in Chemistry Research.

[35] Kononov L O (2015). RSC Adv.

[36] Podvalnyy N M, Malysheva N N, Panova M V, Zinin A I, Chizhov A O, Orlova A V, Kononov L O (2017). Carbohydr Res.

[37] Nagornaya M O, Orlova A V, Stepanova E V, Zinin A I, Laptinskaya T V, Kononov L O (2018). Carbohydr Res.

[38] Myachin I V, Mamirgova Z Z, Stepanova E V, Zinin A I, Chizhov A O, Kononov L O (2022). Eur J Org Chem.

[39] Myachin I V, Kononov L O (2023). Catalysts.

[40] Zhou J, Manabe Y, Tanaka K, Fukase K (2016). Chem – Asian J.

[41] Nagasaki M, Manabe Y, Minamoto N, Tanaka K, Silipo A, Molinaro A, Fukase K (2016). J Org Chem.

[42] Stepanova E V, Podvalnyy N M, Abronina P I, Kononov L O (2018). Synlett.

[43] Jones B, Behm A, Shadrick M, Geringer S A, Escopy S, Lohman M, De Meo C (2019). J Org Chem.

[44] Chen J, Hansen T, Zhang Q-J, Liu D-Y, Sun Y, Yan H, Codée J D C, Schmidt R R, Sun J-S (2019). Angew Chem, Int Ed.

[45] Ogasahara R, Abdullayev S, Sarpe V A, Mandhapati A R, Crich D (2020). Carbohydr Res.

[46] Kononov L O, Fedina K G, Orlova A V, Kondakov N N, Abronina P I, Podvalnyy N M, Chizhov A O (2017). Carbohydr Res.

[47] Hansen T, Lebedel L, Remmerswaal W A, van der Vorm S, Wander D P A, Somers M, Overkleeft H S, Filippov D V, Désiré J, Mingot A (2019). ACS Cent Sci.

[48] Franconetti A, Ardá A, Asensio J L, Blériot Y, Thibaudeau S, Jiménez-Barbero J (2021). Acc Chem Res.

[49] Premathilake H D, Gobble C P, Pornsuriyasak P, Hardimon T, Demchenko A V, De Meo C (2012). Org Lett.

[50] De Meo C, Wallace C E, Geringer S A (2014). Org Lett.

[51] Escopy S, Geringer S A, De Meo C (2017). Org Lett.

[52] Panova M V, Orlova A V, Kononov L O (2018). Russ Chem Bull.

[53] Panova M V, Medvedev M G, Orlova A V, Kononov L O (2022). ChemPhysChem.

[54] Singh P P, Gharia M M, Dasgupta F, Srivastava H C (1977). Tetrahedron Lett.

[55] Stepanova E V, Zinin A I, Abronina P I, Chizhov A O, Kononov L O (2020). Synlett.

[56] Ahiadorme D A, Podvalnyy N M, Orlova A V, Chizhov A O, Kononov L O (2016). Russ Chem Bull.

[57] Orlova A V, Ahiadorme D A, Laptinskaya T V, Kononov L O (2021). Russ Chem Bull.

[58] Orlova A V, Laptinskaya T V, Malysheva N N, Kononov L O (2020). J Solution Chem.

[59] Pazynina G, Tyrtysh T, Nasonov V, Belyanchikov I, Paramonov A, Malysheva N, Zinin A, Kononov L, Bovin N (2013). Synlett.

[60] Orlova A V, Kononov L O (2020). RENSIT.

[61] Kononov L O, Tsvetkov D E, Orlova A V (2002). Russ Chem Bull.

[62] Orlova A V, Andrade R R, da Silva C O, Zinin A I, Kononov L O (2014). ChemPhysChem.

[63] Orlova A V, Zinin A I, Kononov L O (2014). Russ Chem Bull.

[64] Orlova A V, Laptinskaya T V, Bovin N V, Kononov L O (2017). Russ Chem Bull.

[65] Mayr H (2016). Isr J Chem.

[66] Mayr H, Ofial A R (2016). Acc Chem Res.

[67] Lenoir D, Tidwell T T (2018). J Phys Org Chem.

[68] Douglas N L, Ley S V, Lücking U, Warriner S L (1998). J Chem Soc, Perkin Trans 1.

[69] Zhang Z, Ollmann I R, Ye X-S, Wischnat R, Baasov T, Wong C-H (1999). J Am Chem Soc.

[70] Cheng C-W, Zhou Y, Pan W-H, Dey S, Wu C-Y, Hsu W-L, Wong C-H (2018). Nat Commun.

[71] Cheng C-W, Wu C-Y, Hsu W-L, Wong C-H (2020). Biochemistry.

[72] Chang C-W, Lin M-H, Chan C-K, Su K-Y, Wu C-H, Lo W-C, Lam S, Cheng Y-T, Liao P-H, Wong C-H (2021). Angew Chem, Int Ed.

[73] Chatterjee S, Moon S, Hentschel F, Gilmore K, Seeberger P H (2018). J Am Chem Soc.

[74] Moon S, Chatterjee S, Seeberger P H, Gilmore K (2021). Chem Sci.

[75] Kancharla P K, Crich D (2013). J Am Chem Soc.

[76] Dharuman S, Amarasekara H, Crich D (2018). J Org Chem.

[77] Ngoje P, Crich D (2020). J Am Chem Soc.

[78] Upadhyaya K, Bagul R S, Crich D (2021). J Org Chem.

[79] Jeanneret R A, Johnson S E, Galan M C (2020). J Org Chem.

[80] Siyabalapitiya Arachchige S, Crich D (2022). J Org Chem.

