# Supplementary material for: Comparison of glycosyl donors: a supramer approach
Source: Beilstein J Org Chem. 2024 Jan 31;20:181–92. doi: 10.3762/bjoc.20.18 (PMC10840533; doi:10.3762/bjoc.20.18)
Supplement: File 1 — Copies of NMR spectra for all new compounds, single crystal X-ray analysis data for compound 7 (CCDC 1843708). [file Beilstein_J_Org_Chem-20-181-s001.pdf]

BEILSTEIN JOURNAL OF ORGANIC CHEMISTRY

Supporting Information

for

Comparison of glycosyl donors: a supramer approach

Anna V. Orlova, Nelly N. Malysheva, Maria V. Panova, Nikita M. Podvalnyy, Michael G. Medvedev and Leonid O. Kononov

Beilstein J. Org. Chem. **2024,** *20,* 181–192. doi:10.3762/bjoc.20.18

Copies of NMR spectra for all new compounds, single crystal X-ray analysis data for compound 7 (CCDC 1843708)

License and Terms: This is a supporting information file under the terms of the Creative Commons Attribution License (https://creativecommons.org/licenses/by/4.0). Please note that the reuse, redistribution and reproduction in particular requires that the author(s) and source are credited and that individual graphics may be subject to special legal provisions.
The license is subject to the *Beilstein Journal of Organic Chemistry* terms and conditions: (https://www.beilstein-journals.org/bjoc/terms)

Table of contents

Single crystal X-ray analysis of compound **7** S2

Table S1. Results of sialylation with sialyl donors **1** and **2** at 0.05 M and 0.15 M concentrations S3

Figure S1. A part of ^{1}H NMR spectrum of disaccharide fraction (Entry 1 in Table 1S) S3

Figure S2. A part of ^{1}H NMR spectrum of disaccharide fraction (Entry 2 in Table 1S) S4

Figure S3. A part of ^{1}H NMR spectrum of disaccharide fraction (Entry 3 in Table 1S) S4

Figure S4. A part of ^{1}H NMR spectrum of disaccharide fraction (Entry 4 in Table 1S) S5

Figure S5. The general view of a molecule of **7** in a crystal S6

Figure S6. Superposition of the main conformations of the three independent molecules of **7** in a crystal S7

Figure S7. The general view of the disordered molecule of **7** in a crystal S8

Figure S8. Infinite helixes formed in the crystal structure of **7** S9

NMR spectra of compound **6** S10

NMR spectra of compound **7** S15

NMR spectra of compound **2** S21

NMR spectra of compound **α-4** S24

References S26

Single crystal X-ray analysis of methyl (phenyl 3,5-dideoxy-2-thio-4,9-bis-*O*-chloroacetyl-5-trifluoroacetamido-D-glycero-β-D-galacto-nonulopyranosid)onate (7)

Single crystals of methyl (phenyl 3,5-dideoxy-2-thio-4,9-bis-*O*-chloroacetyl-5-trifluoroacetamido-D-glycero-β-D-galacto-nonulopyranosid)onate water solvate (**7**·H_2O) were grown from acetone-d_6 in an NMR tube. A suitable crystal was selected and the XRD analysis was performed on a Bruker SMART APEX2 DUO CCD diffractometer (Cu $K_α$ radiation, λ = 1.54178 Å). The crystal was kept at 120 K during data collection. The data set was corrected for absorption with SADABS [1], the structure was solved with the ShelXT [2,3] structure solution program using Direct Methods and refined with the ShelXL [2,3] refinement package using Least Squares minimization (Olex2 [4] was used as an interface to ShelXT and ShelXL packages). Non-hydrogen atoms were refined anisotropically. The hydrogen atoms of water molecules, amino and hydroxy groups were localized in the difference-Fourier maps and refined isotropically with X-H distances fixed at values 0.84 Å for O–H and 0.95 Å for N–H bonds. The other hydrogen atoms were placed in calculated positions and refined within riding model with fixed isotropic displacement parameters [Uiso(H) = 1.2 Ueq(C)].

Crystal Data for $6C_{22}H_{24}Cl_2F_3NO_{10}S·3H_2O$ [$C_{132}H_{150}Cl_{12}F_{18}N_6O_{63}S_6$] ($M$ = 3788.23 g·mol^{-1}): hexagonal, space group $P6_1$ (no. 169), a = 22.9311(10) Å, c = 27.2831(12) Å, V = 12424.4(12) Å^3, Z = 3, T = 120 K, μ(CuKα) = 3.520 mm^{-1}, D_{calc} = 1.519 g·cm^{-3}, 78997 reflections measured (4.45° ≤ 2Θ ≤ 146.052°), 15162 unique (R_{int} = 0.0838, R_{sigma} = 0.0608) which were used in all calculations. The final R_1 was 0.0531 (I > 2σ(I)) and wR_2 was 0.1498 (all data). Crystal structure has been deposited with Cambridge Crystallographic Data Center (CCDC reference number 1843708).

Crystal unit of methyl (phenyl 3,5-dideoxy-2-thio-4,9-bis-*O*-chloroacetyl-5-trifluoroacetamido-D-glycero-β-D-galacto-nonulopyranosid)onate (further, carbohydrate) water solvate constitutes of three independent carbohydrate molecules and 1.5 water molecules. The main conformation of one of the carbohydrate molecules is shown in Figure S5. A superposition of the main conformations of all three independent carbohydrate molecules is shown in Figure S6.

Two of the carbohydrate molecules are well ordered (with slightly disordered CH_2Cl, CF_3 and Ph groups) and have very similar conformations (Figure S5). They are connected by hydrogen bonds (both direct and through a complete water molecule) to form an infinite helix (Helix 1 on Figure S8). The period of this helix is 27.2831(12) Å and consists of 6 carbohydrate molecules.

The third carbohydrate molecule is strongly disordered (Figure S7, shift between parts is about 0.55–0.75 Å) with identical occupancies of both parts. This molecule has a conformation different from the first two (Figures S6, S7). It is bonded by hydrogen bonds to its symmetrical images in neighboring cells to form another infinite helix (Helix 2 on Figure S8). The period of this helix is identical to that of the first helix (27.2831(12) Å) and also consists of 6 carbohydrate molecules. Given the identical occupancies of the disordered parts, it is likely that they alternate in the helix.

Overall, the crystal structure consists of two independent infinite helixes, and a disordered halfly occupied water molecules residing between them.

Table S1. Conditions and results of sialylation of glycosyl acceptor **3** with sialyl donors **1** and **2** at 0.05 and 0.15 mol·L^{-1} concentrations (*c*).

entry	sialyl donor	R	*c* (mol·L^{-1})	α/β	yield (%)	time (h)
1[a]	**1**	TFA	0.05	16:1	55	2.5
2	**1**	TFA	0.15	12:1	66	3
3	**2**	ClAc	0.05	13:1	57	2
4	**2**	ClAc	0.15	18:1	62	1

[a] Data taken from [5].

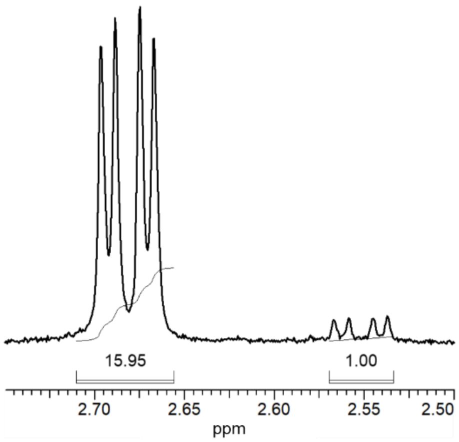

Figure S1. A part of ^{1}H NMR spectrum of disaccharide fraction (Entry 1 in Table S1) showing the signals of α-H-3eq and β-H-3eq of Neu5Ac residue of **4**.

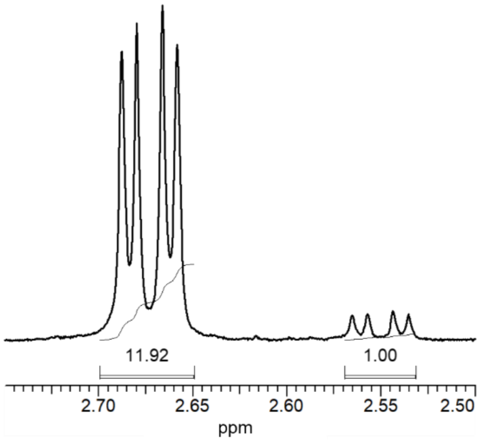

Figure S2. A part of ^{1}H NMR spectrum of disaccharide fraction (Entry 2 in Table S1) showing the signals of α-H-3eq and β-H-3eq of Neu5Ac residue of **4**.

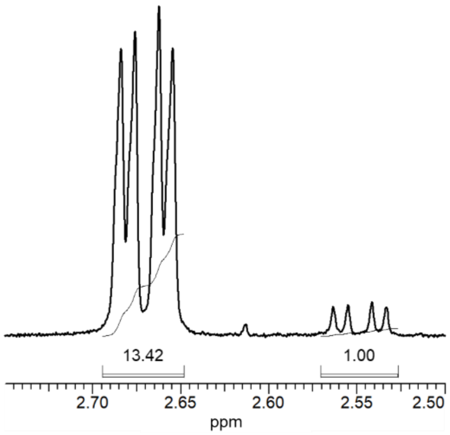

Figure S3. A part of ^{1}H NMR spectrum of disaccharide fraction (Entry 3 in Table S1) showing the signals of α-H-3eq and β-H-3eq of Neu5Ac residue of **4**.

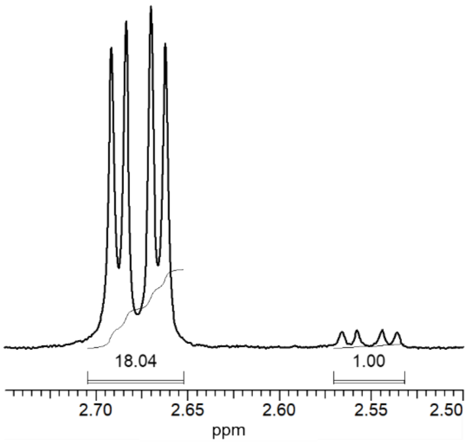

Figure S4. A part of ^{1}H NMR spectrum of disaccharide fraction (Entry 4 in Table S1) showing the signals of α-H-3eq and β-H-3eq of Neu5Ac residue of **4**.

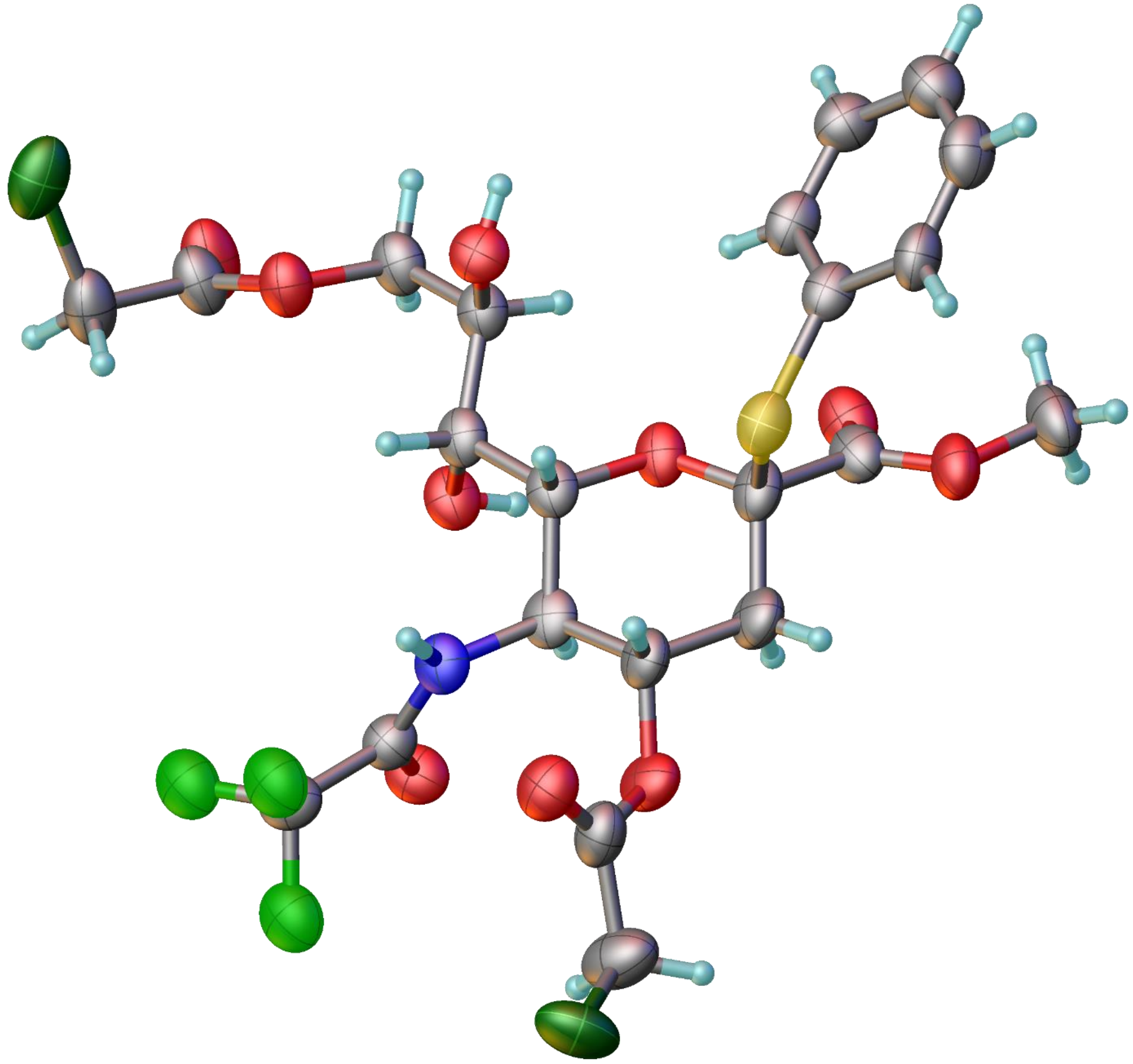

Figure S5. The general view of a molecule of **7** in the crystal in representation of atoms by thermal ellipsoids (*p* = 40%). Coloring: carbon – grey, oxygen – red, nitrogen – blue, fluorine – light green, sulphur – yellow, chlorine – dark green, hydrogen – sky blue.

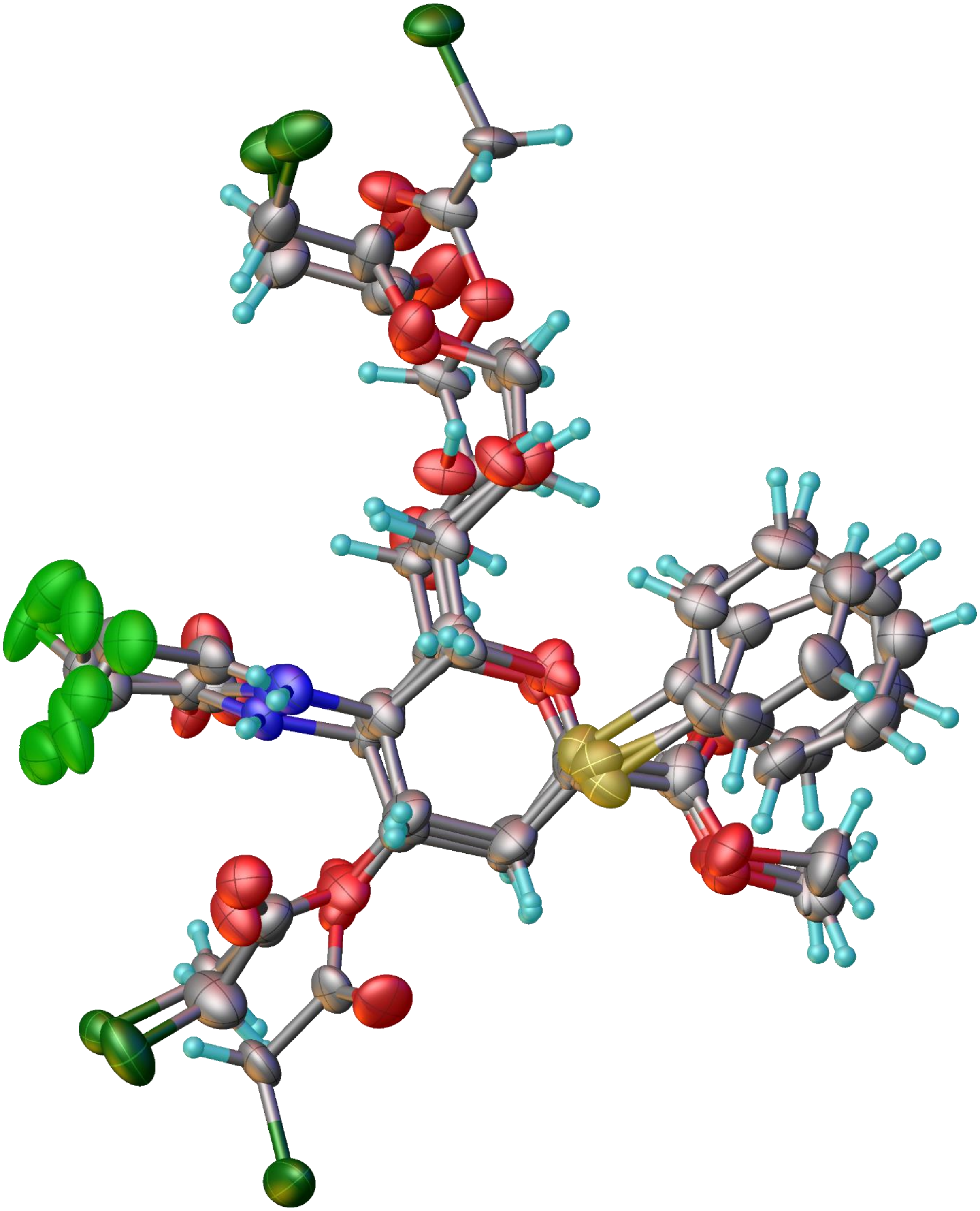

Figure S6. Superposition of the main conformations of the three independent molecules of **7** in the crystal in representation of atoms by thermal ellipsoids (p = 40%). Coloring: carbon – grey, oxygen – red, nitrogen – blue, fluorine – light green, sulphur – yellow, chlorine – dark green, hydrogen – sky blue.

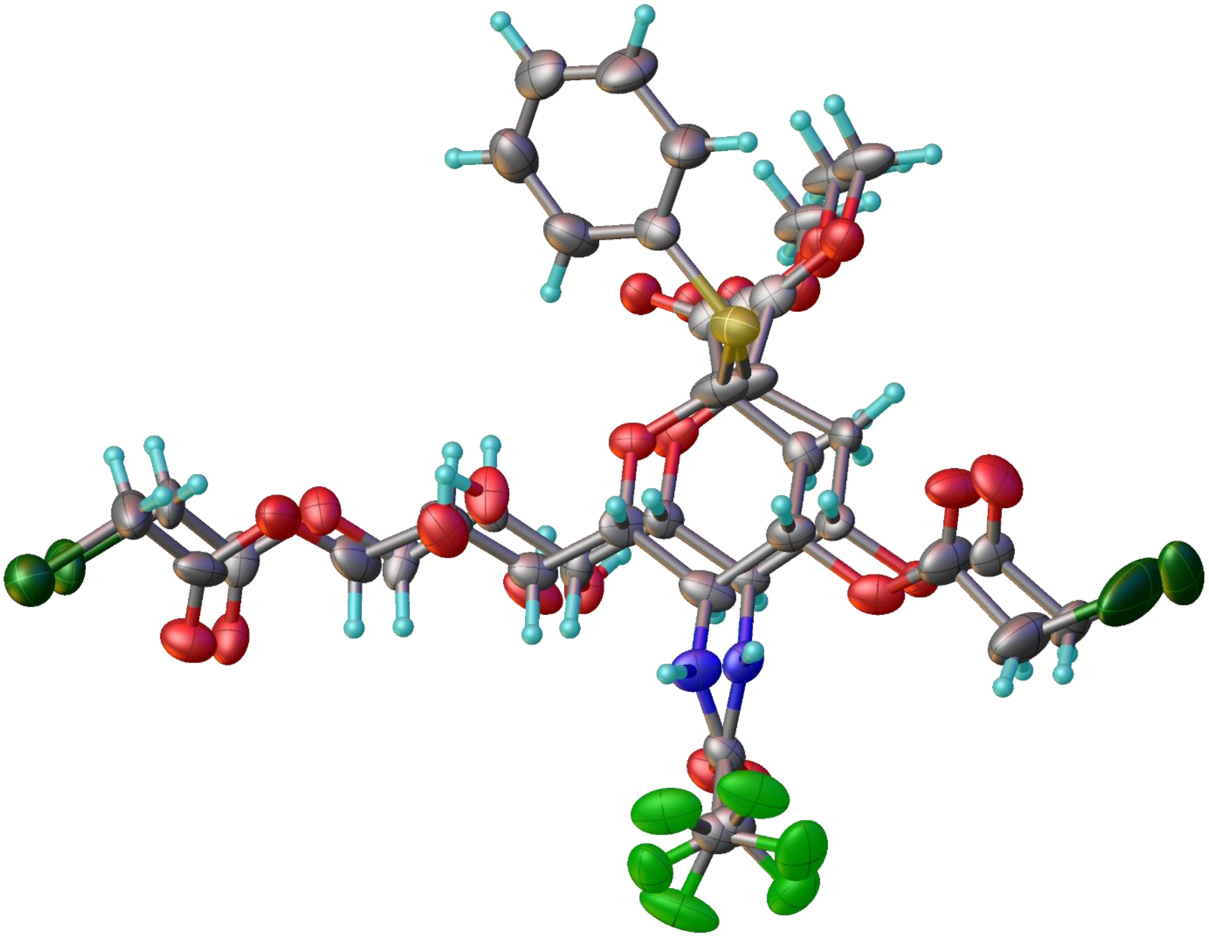

Figure S7. The general view of the disordered molecule of **7** in the crystal in representation of atoms by thermal ellipsoids (*p* = 40%). Coloring: carbon – grey, oxygen – red, nitrogen – blue, fluorine – light green, sulphur – yellow, chlorine – dark green, hydrogen – sky blue.

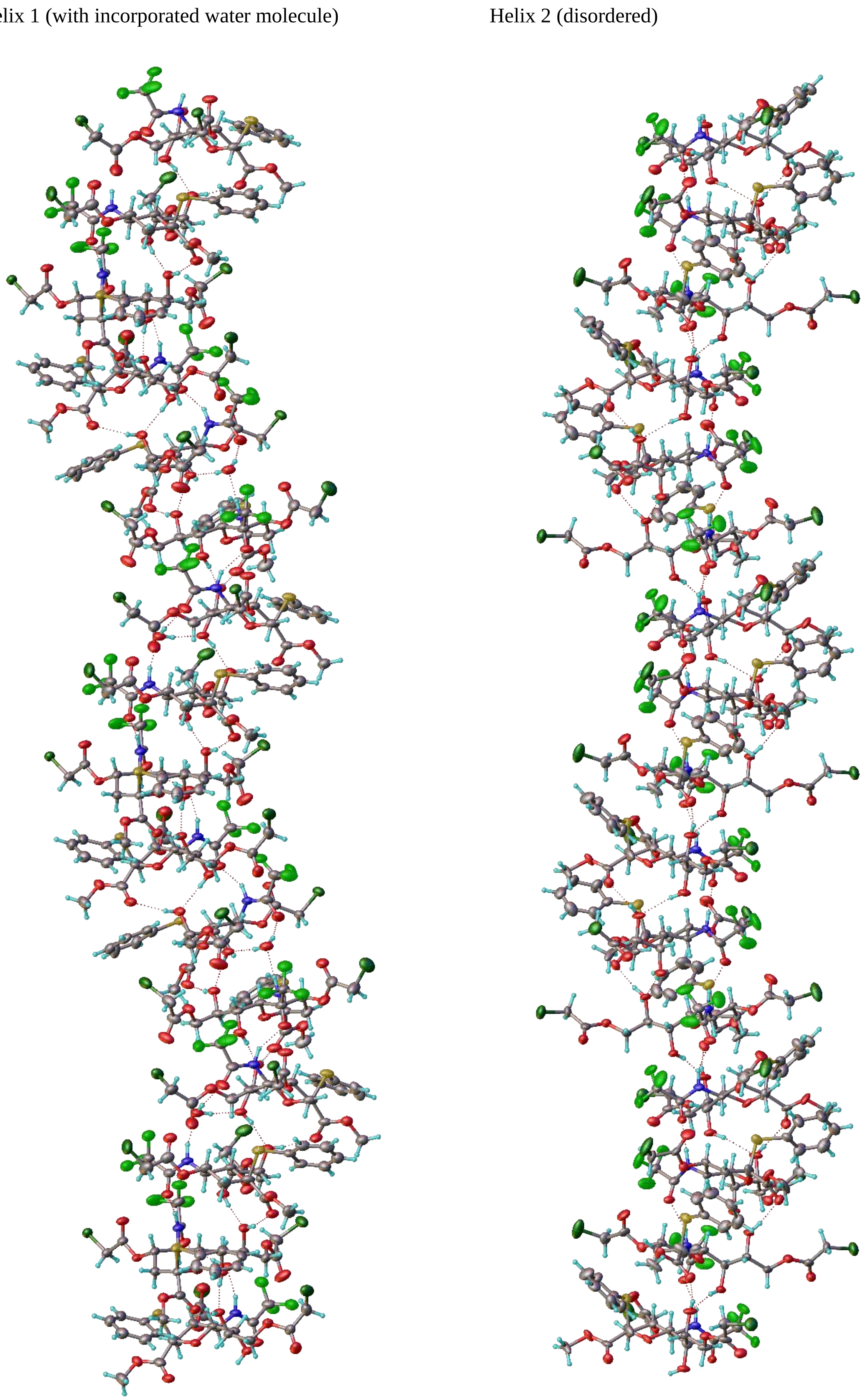

Figure S8. Infinite helixes formed in the crystal structure of **7**. Atoms are represented by thermal ellipsoids (p = 40%). Coloring: carbon – grey, oxygen – red, nitrogen – blue, fluorine – light green, sulphur – yellow, chlorine – dark green, hydrogen – sky blue.

^{1}H NMR (300 MHz) spectrum of compound 6 in $CDCl_3$

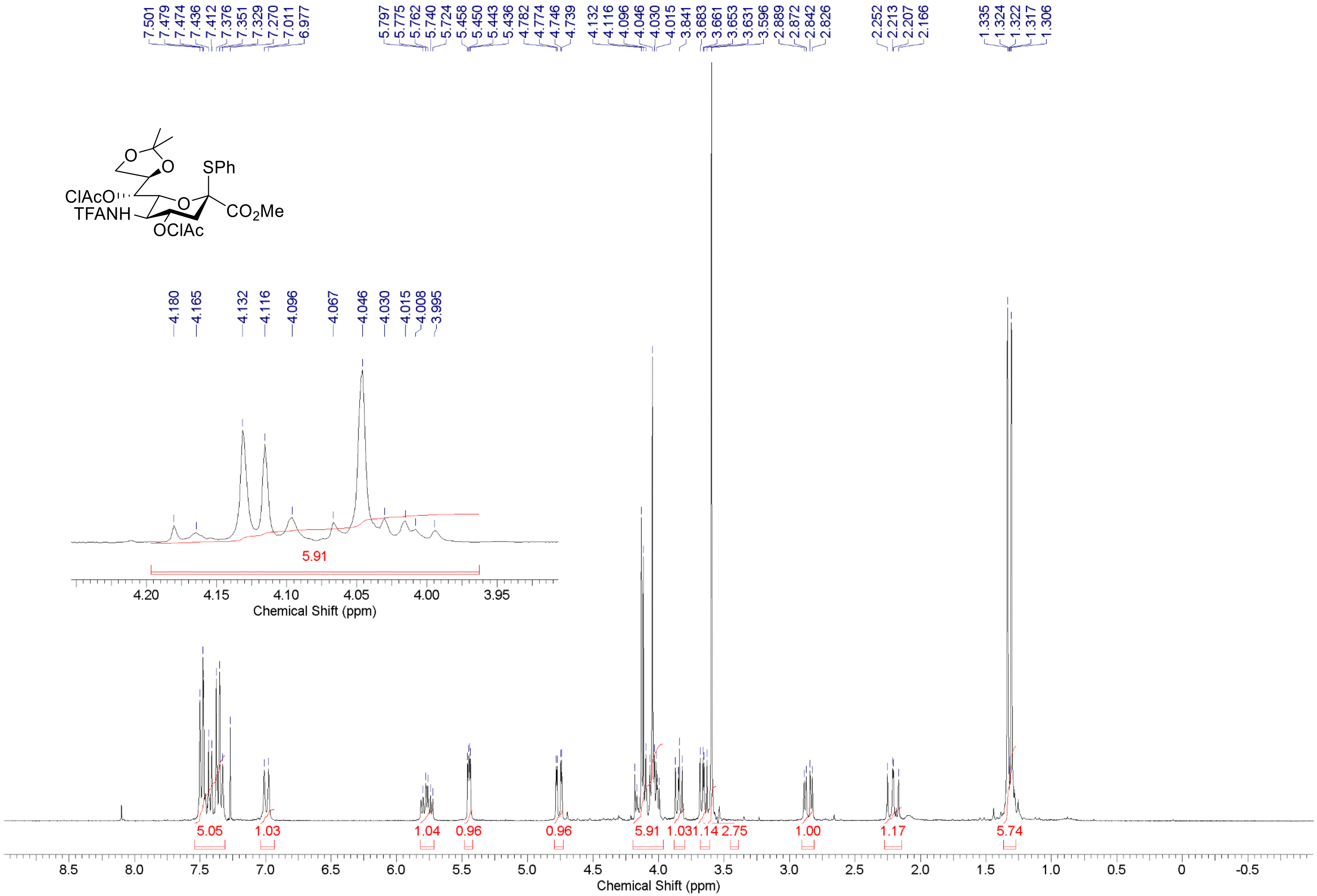

^{13}C NMR (75 MHz) spectrum of compound 6 in $CDCl_3$

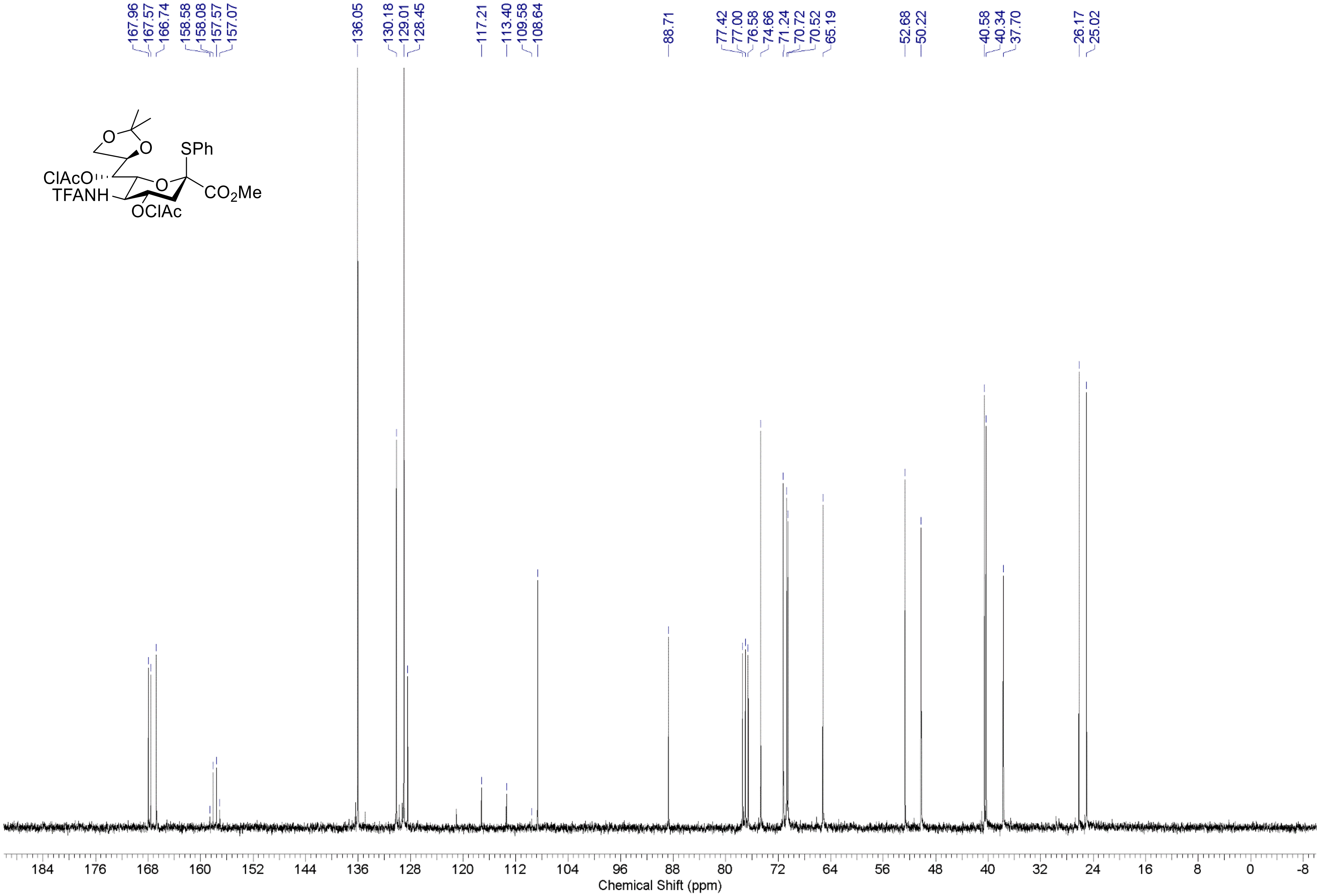

^{19}F NMR (282 MHz) spectrum of compound 6 in $CDCl_3$

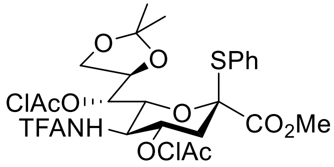

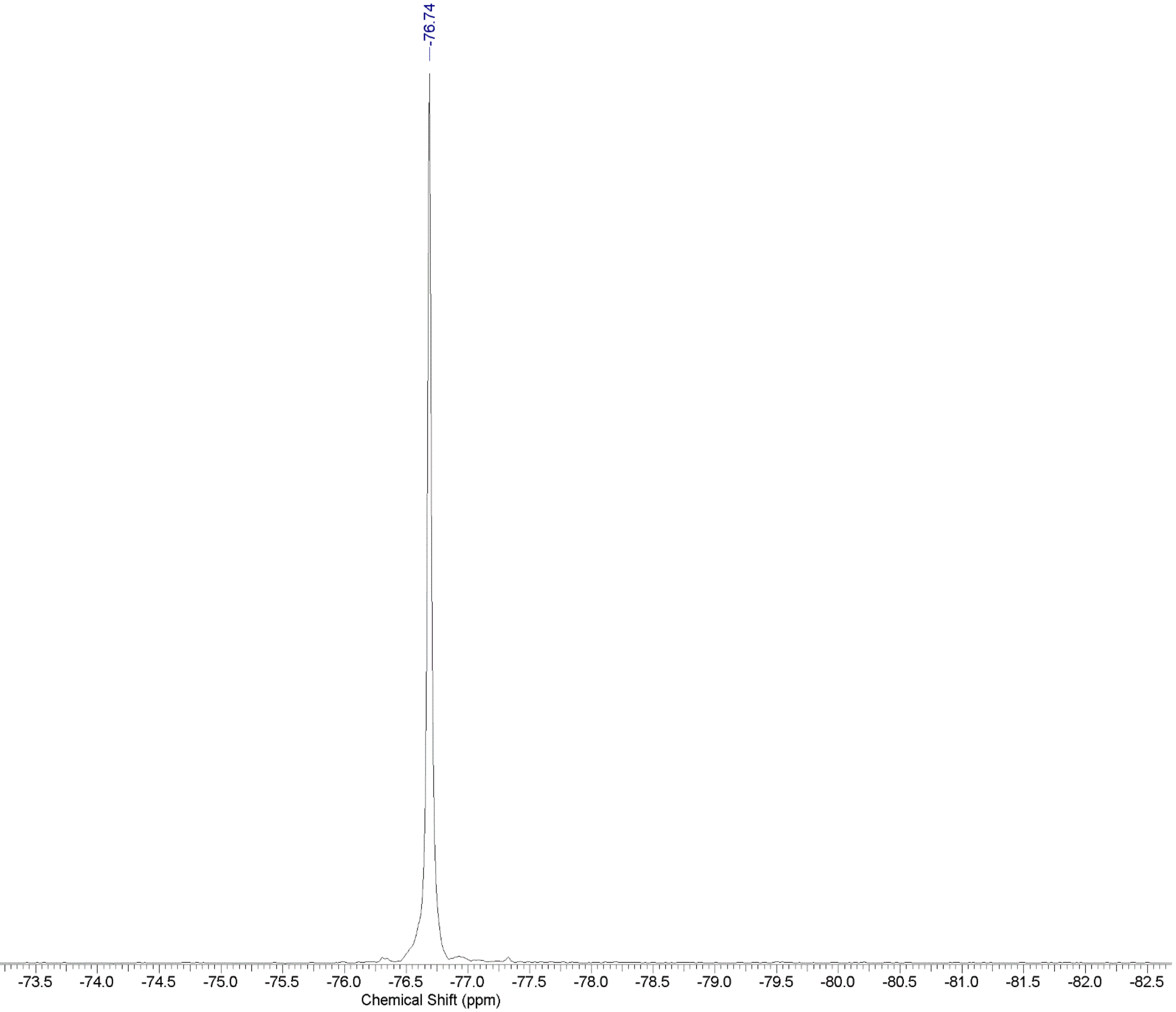

COSY (300 MHz) spectrum of compound 6 in $CDCl_3$

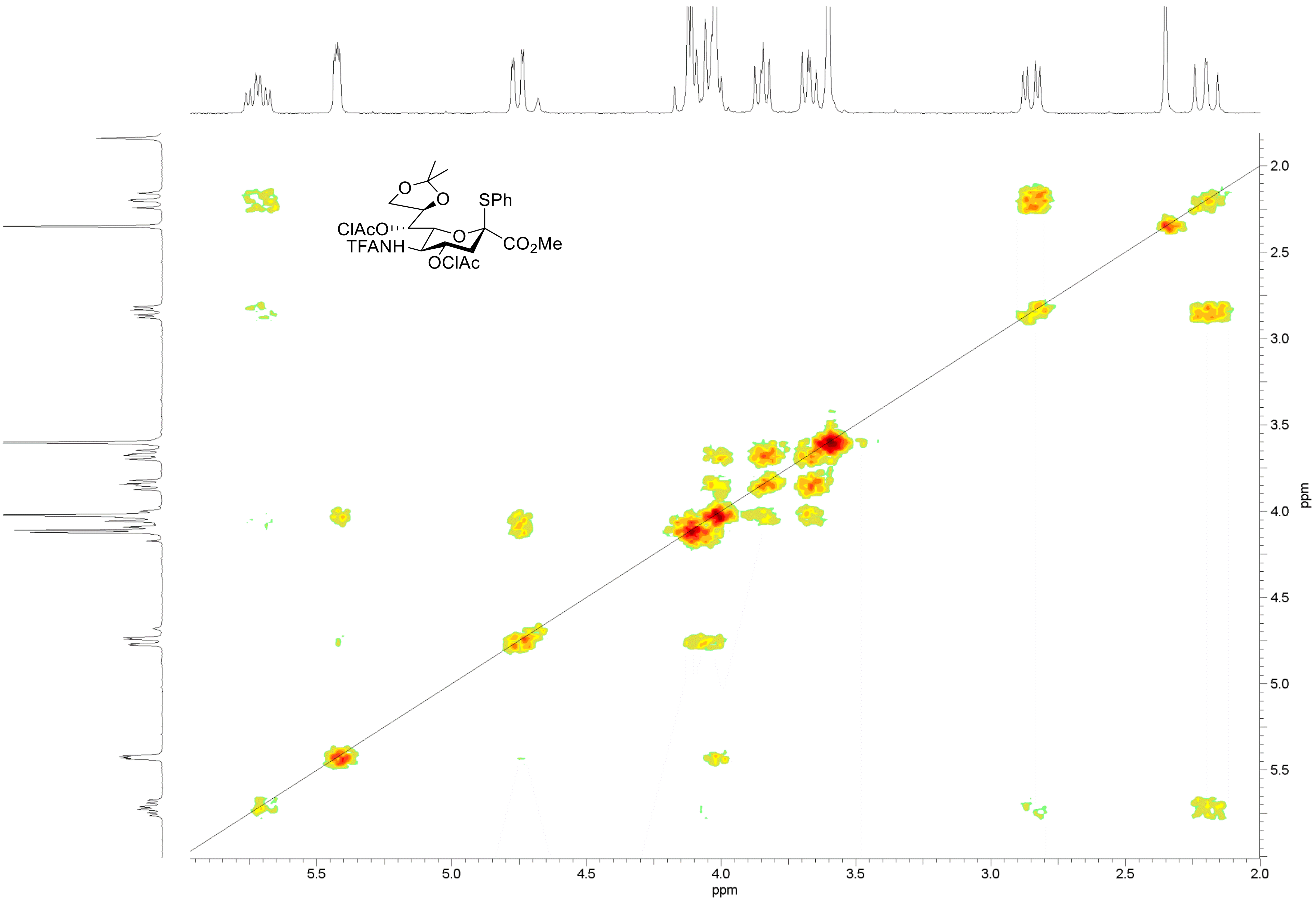

HSQC (300 MHz) spectrum of compound 6 in $CDCl_3$

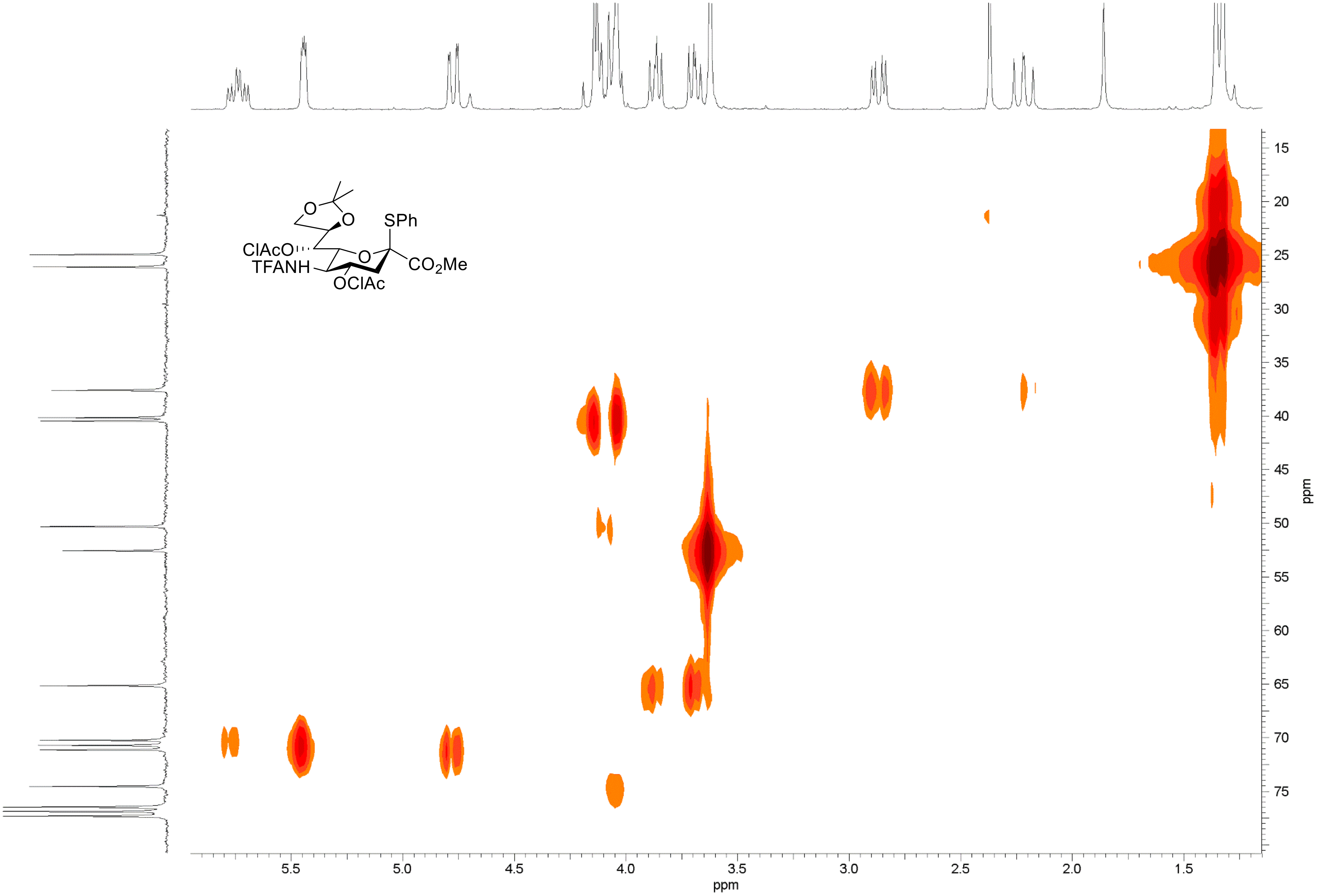

^1H NMR (600 MHz) spectrum of compound 7 in acetone-d_6

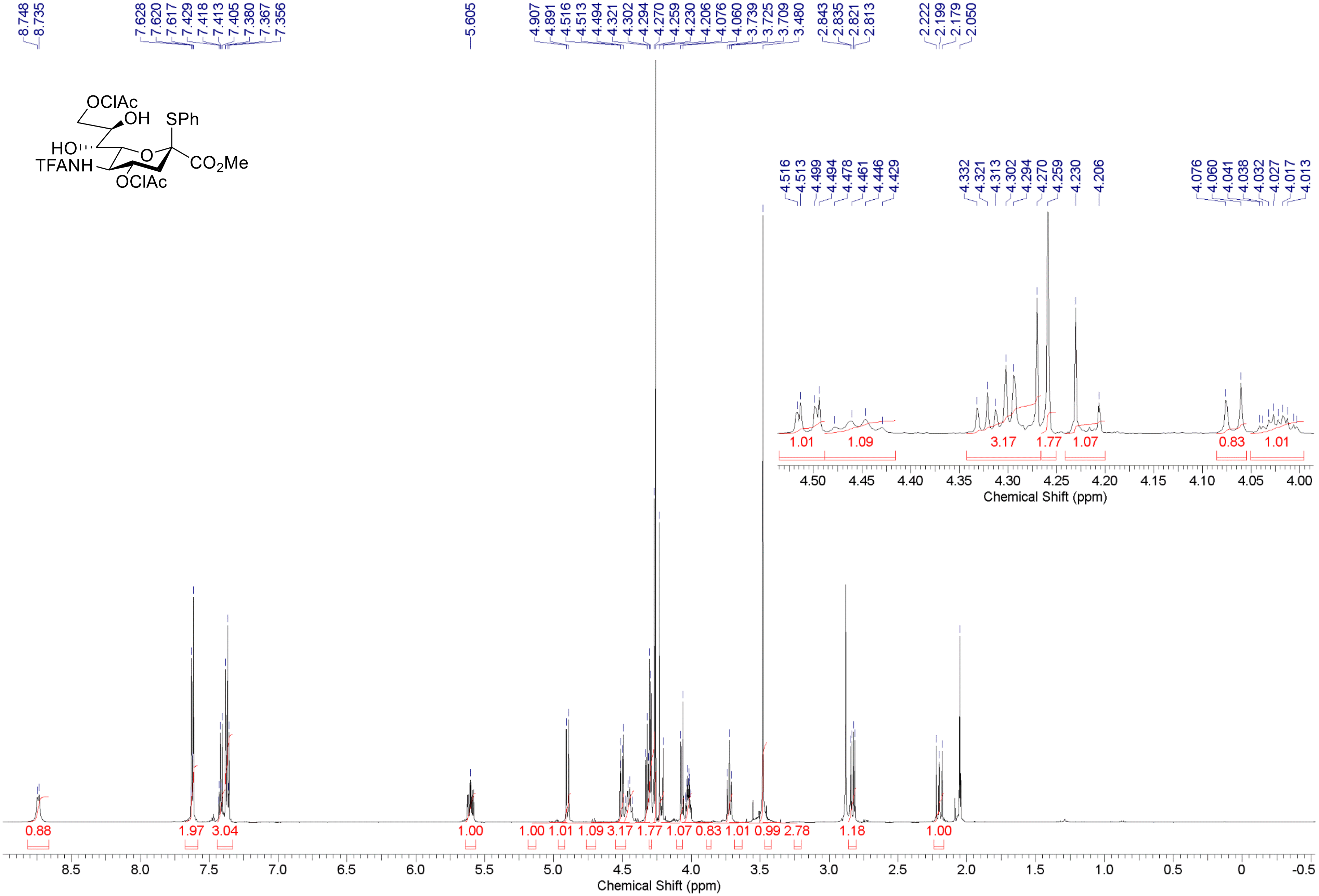

^{13}C NMR (151 MHz) spectrum of compound 7 in acetone-d_6

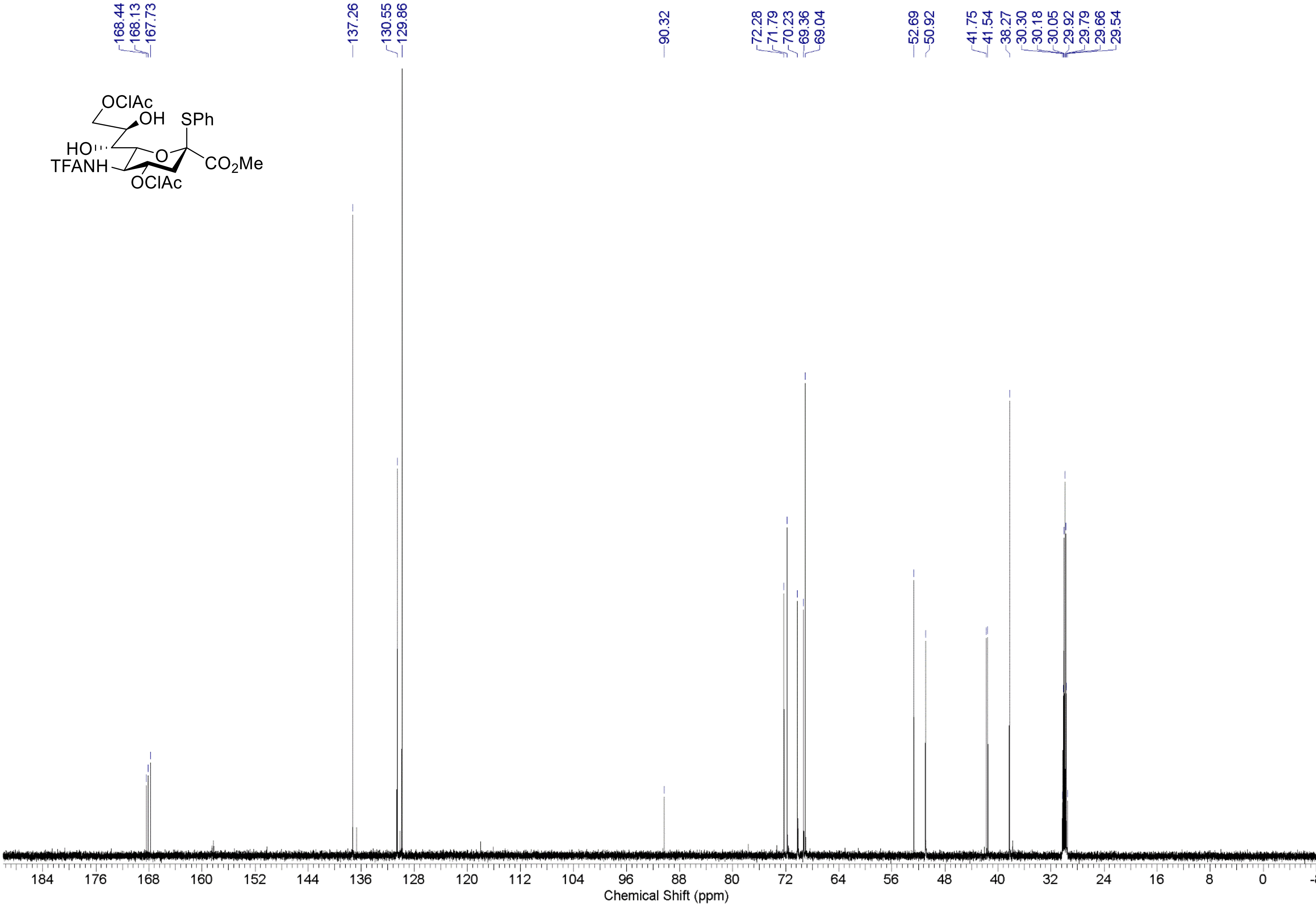

^{19}F NMR (282 MHz) spectrum of compound 7 in acetone-d_6

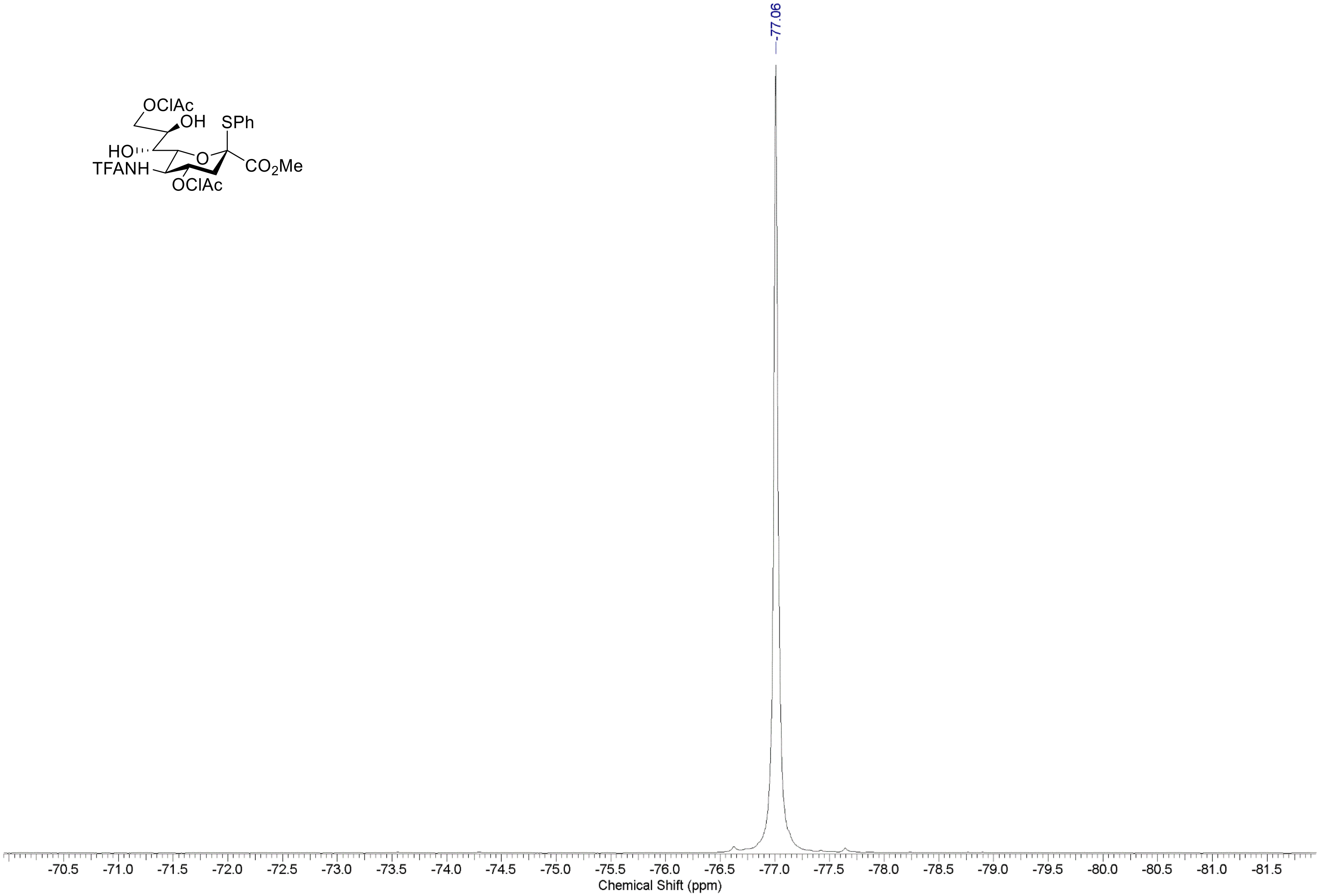

COSY (600 MHz) spectrum of compound 7 in acetone-d_6

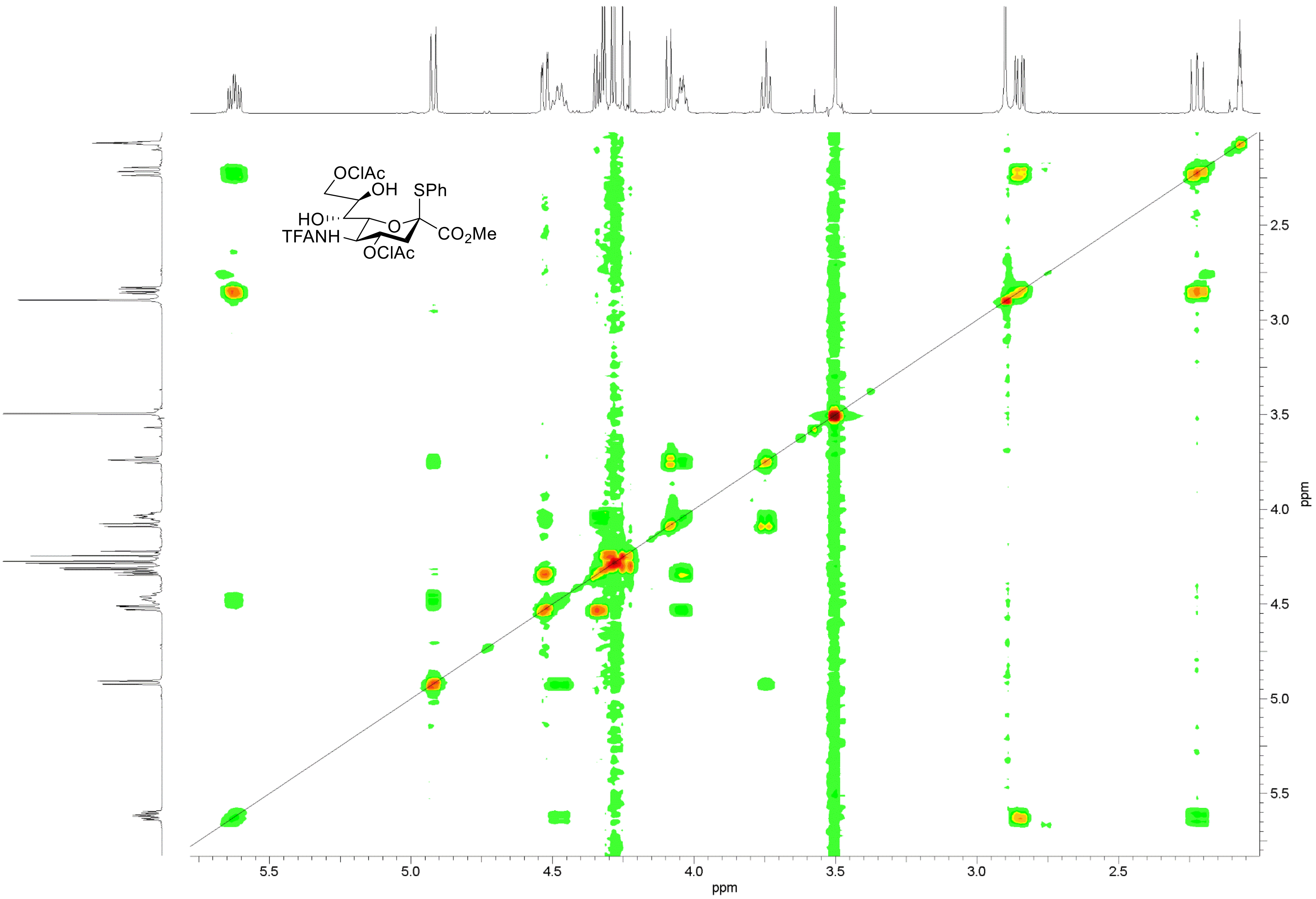

HSQC (600 MHz) spectrum of compound 7 in acetone-d_6

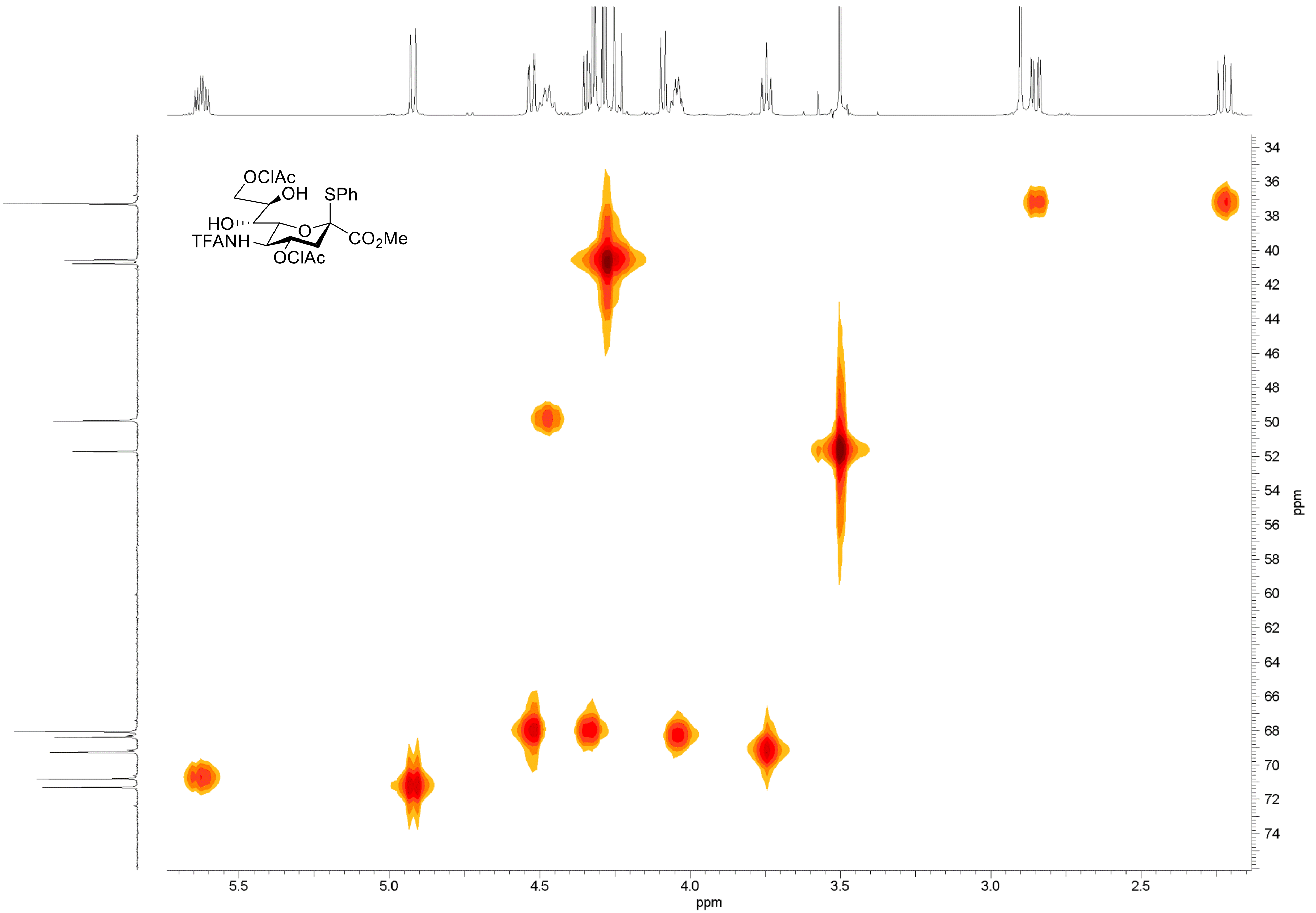

HMBC (600 MHz) spectrum of compound 7 in acetone-d_6

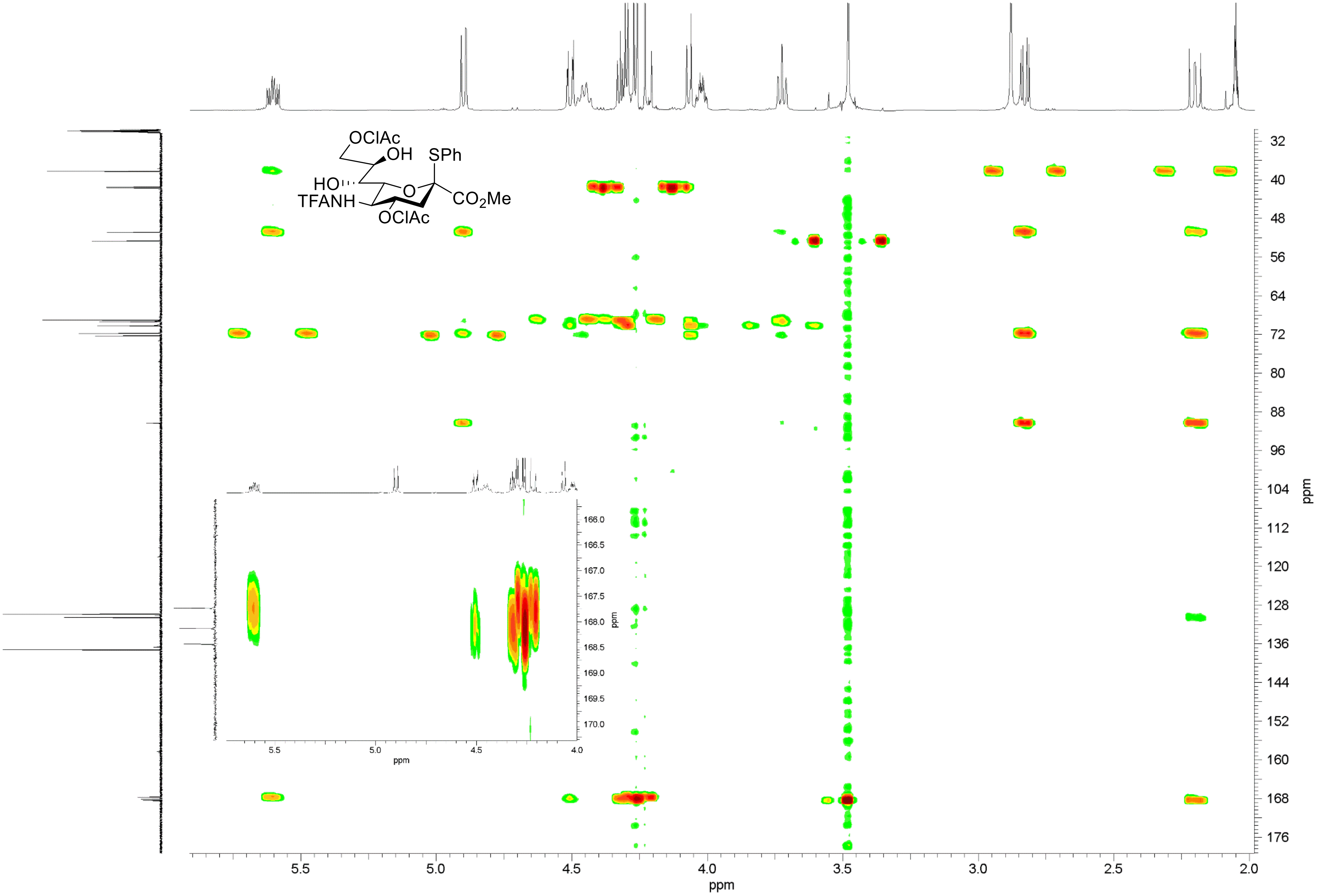

^{1}H NMR (300 MHz) spectrum of compound 2 in $CDCl_3$

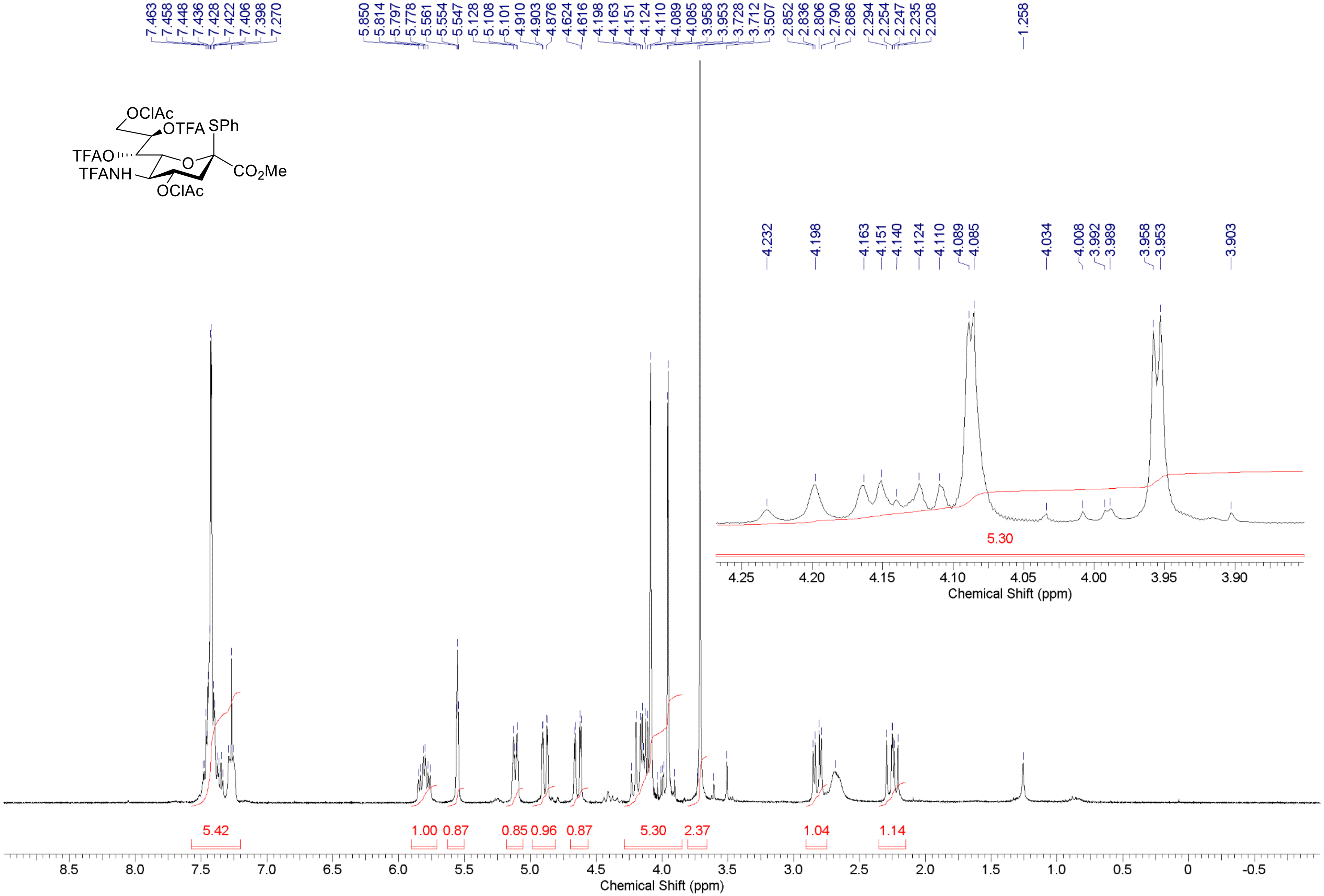

^{13}C NMR (75 MHz) spectrum of compound 2 in $CDCl_3$

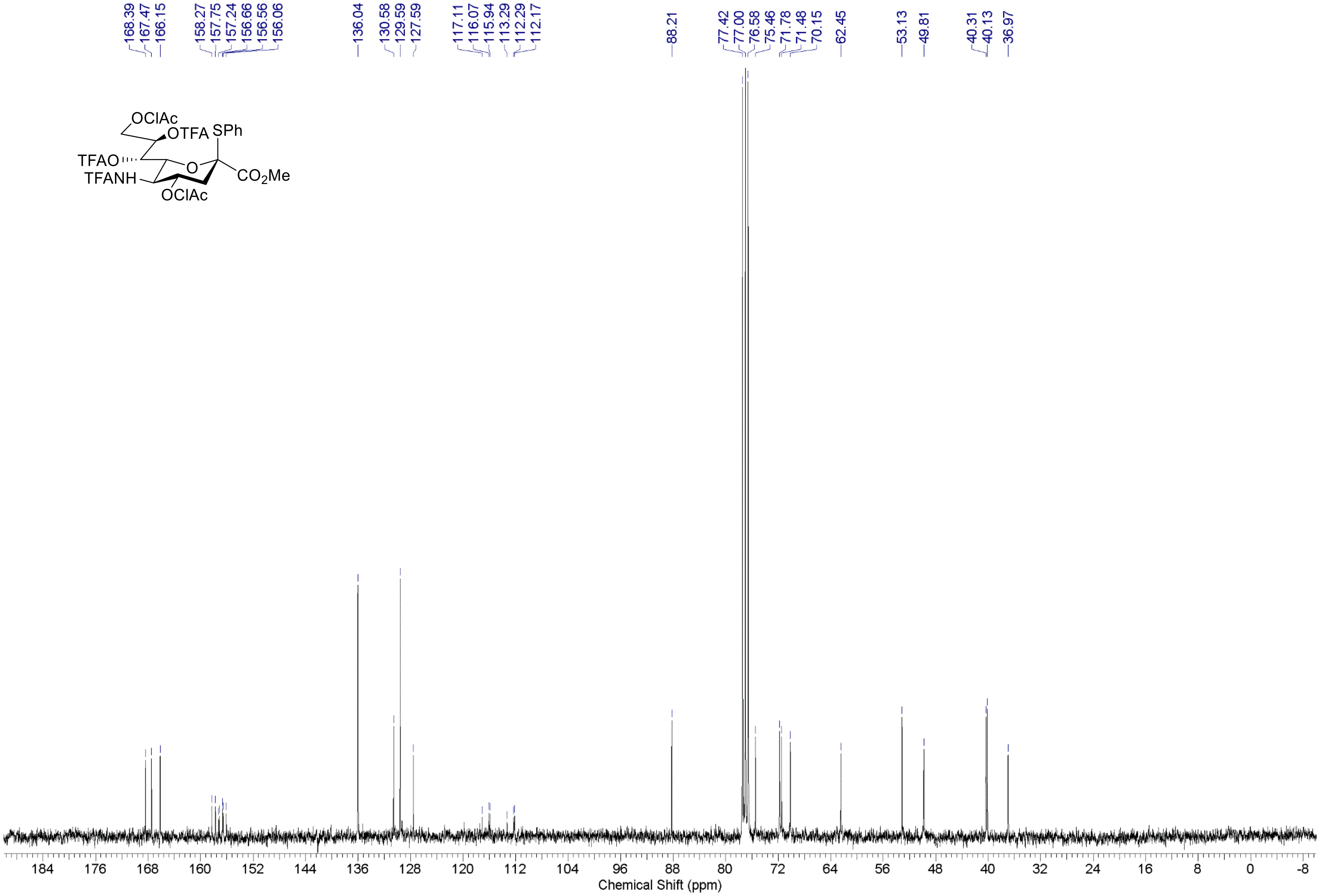

^{19}F NMR (282 MHz) spectrum of compound 2 in $CDCl_3$

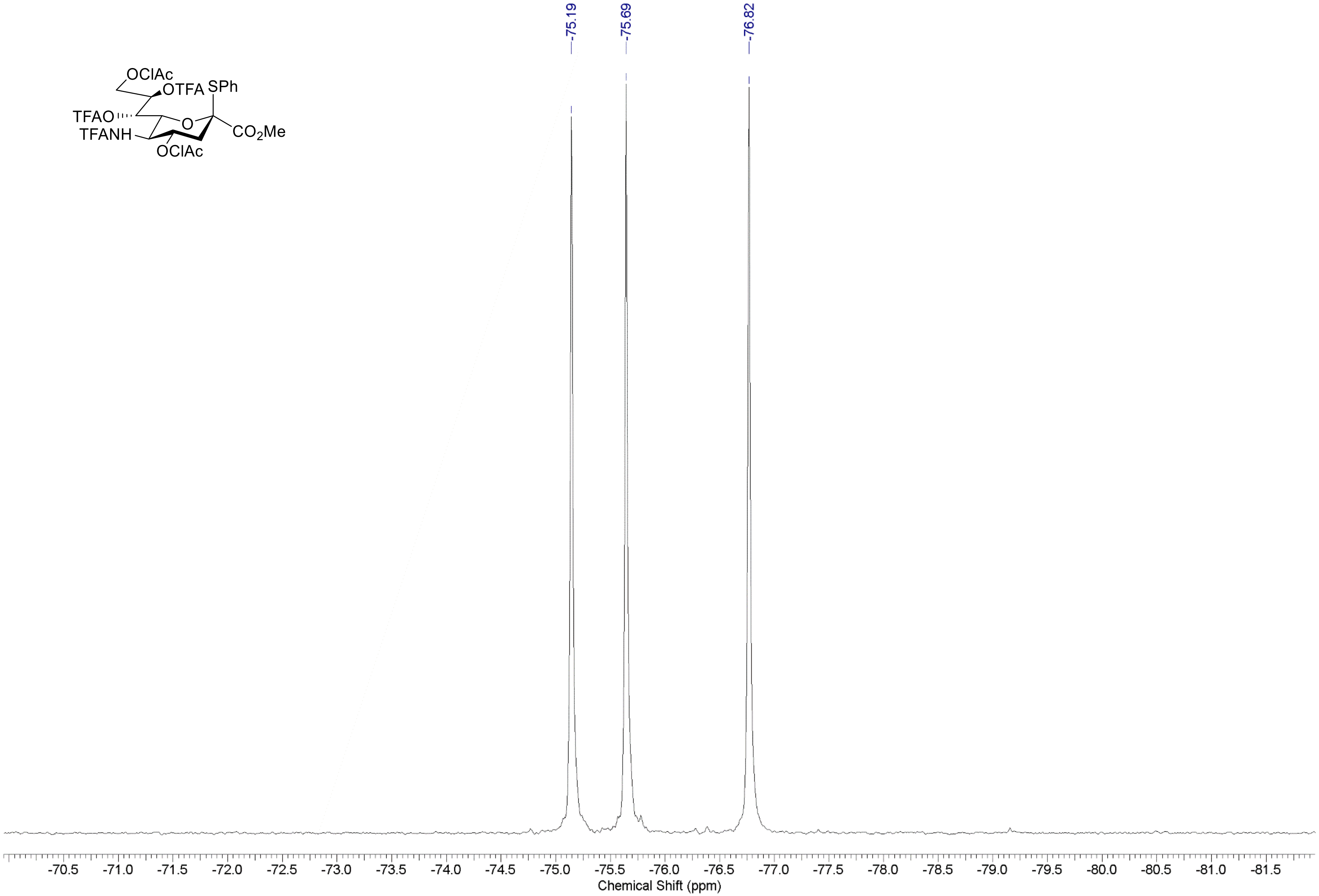

^{1}H NMR (600 MHz) spectrum of compound α-4 in $CDCl_3$

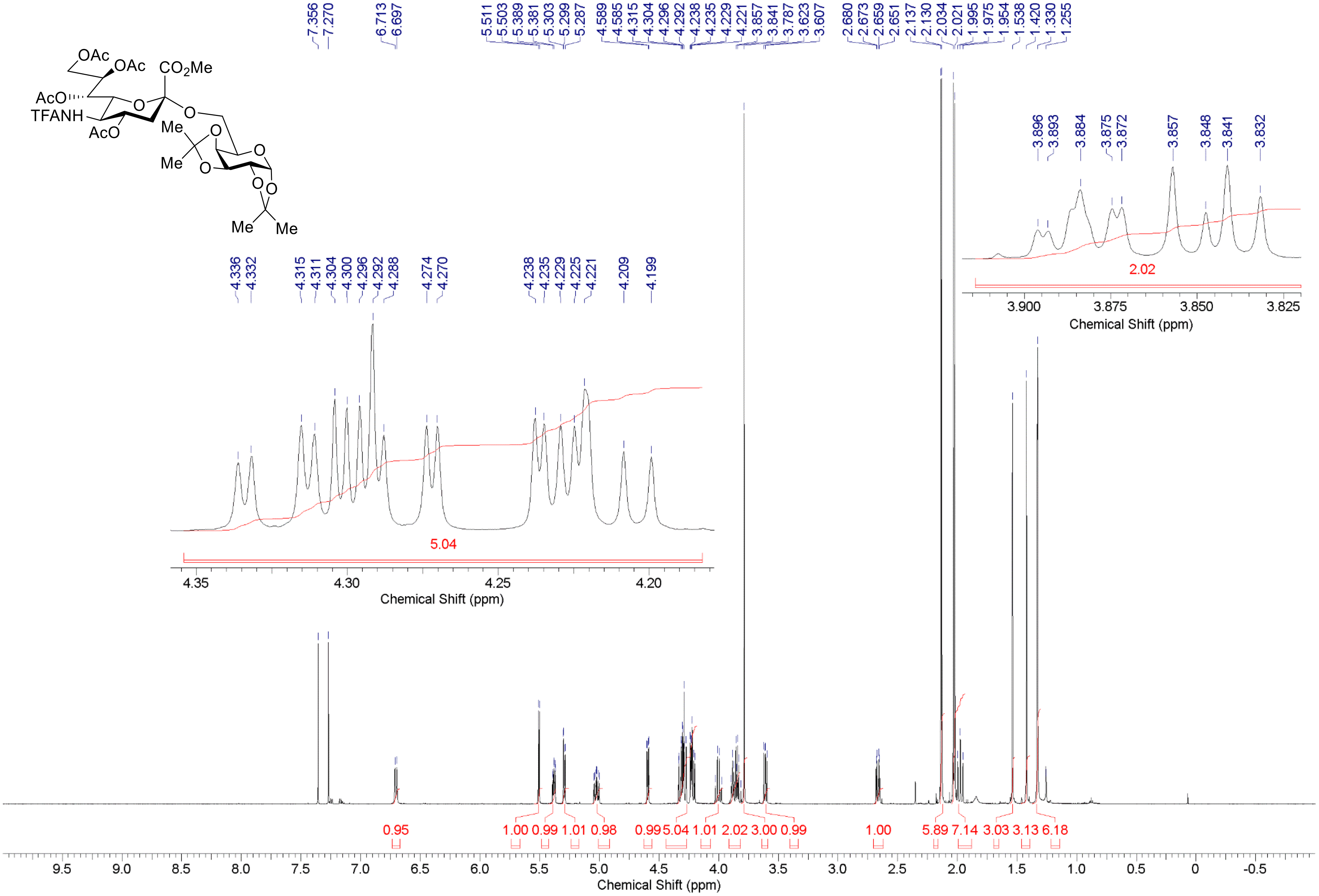

^{13}C NMR (151 MHz) spectrum of compound α-4 in $CDCl_3$

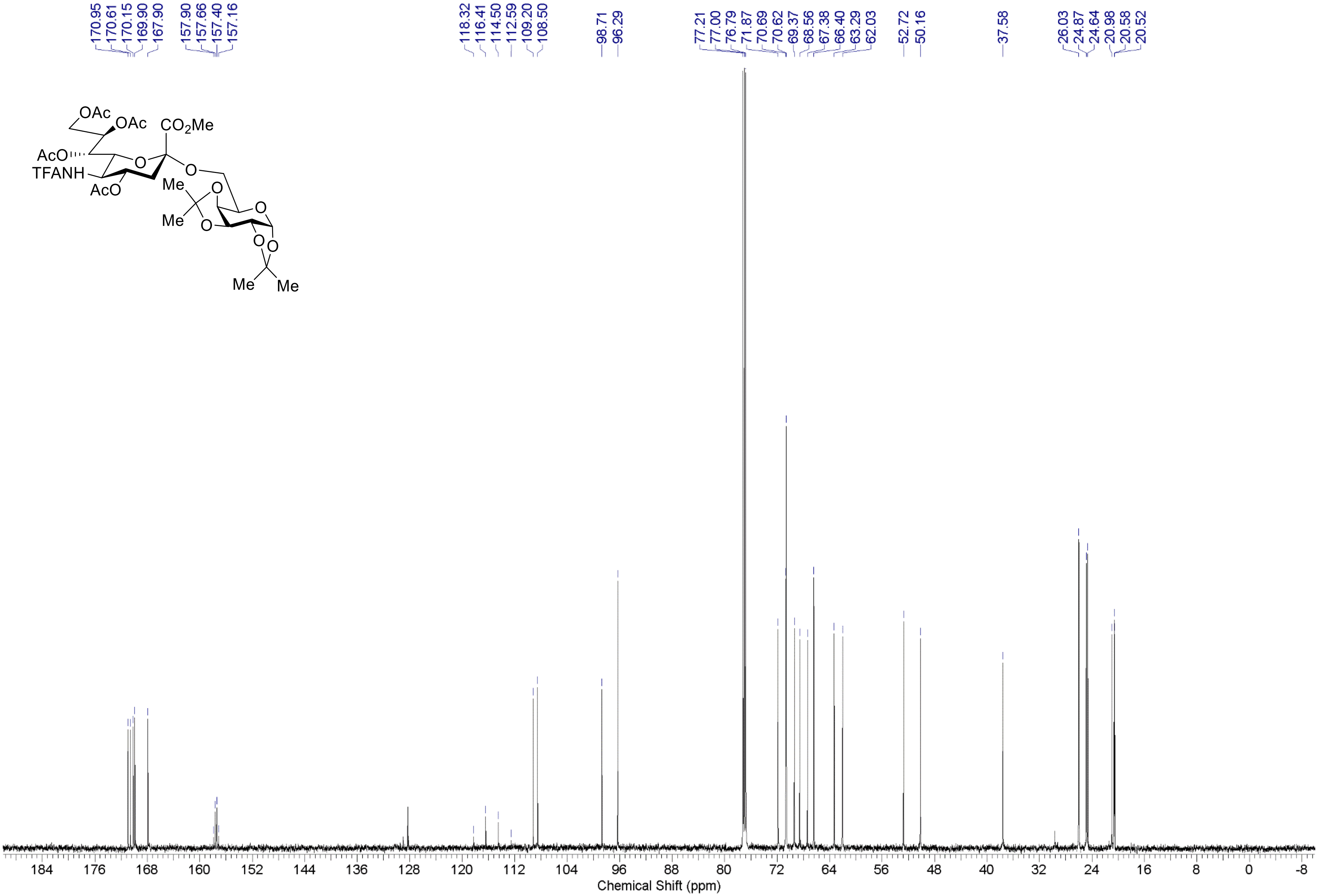

References

1. Sheldrick G. M., SADABS, v. 2.03, Bruker/Siemens Area Detector Absorption Correction Program; Bruker AXS Inc., Madison, WI, 2003
2. Sheldrick, G.M. *Acta Cryst.*, **2015**, *A71*, 3–8.
3. Sheldrick G. M. *Acta Cryst.*, **2008**, *A64*, 112–122.
4. Dolomanov, O. V., Bourhis, L. J.; Gildea, R. J., Howard, J. A. K.; Puschmann, H. *J. Appl. Cryst.*, **2009**, *42*, 339–341.
5. Podvalnyy, N. M.; Malysheva, N. N.; Panova, M. V.; Zinin, A. I.; Chizhov, A. O.; Orlova, A. V.; Kononov, L. O. *Carbohydr. Res.* **2017**, *451*, 12-28 (indentical to the reference in the main article – doi: 10.1016/j.carres.2017.09.002).
