# Supplementary material for: Comparison of glycosyl donors: a supramer approach
Source: Beilstein J Org Chem. 2024 Jan 31;20:181–92. doi: 10.3762/bjoc.20.18 (PMC10840533; doi:10.3762/bjoc.20.18)
Supplement: File 2 — Deposed crystallographic information file (CCDC 1843708) and check file. [file Beilstein_J_Org_Chem-20-181-s002.zip › 1843708_PM02014B-data_sads_h_file002.html]

checkCIF/PLATON report


```
No syntax errors found.                               CIF dictionary  
Please wait while processing ....                     Interpreting this report
```

**Datablock: sads\_h**


---

|  |  |  |
| --- | --- | --- |
| Bond precision: | C-C = 0.0124 A | Wavelength=1.54178 |

|  |  |  |  |
| --- | --- | --- | --- |
| Cell: | a=22.9311(10) | b=22.9311(10) | c=27.2831(12) |
|  | alpha=90 | beta=90 | gamma=120 |
| Temperature: | 120 K |  |  |

|  |  |  |
| --- | --- | --- |
|  | Calculated | Reported |
| Volume | 12424.4(14) | 12424.4(12) |
| Space group | P 61 | P 61 |
| Hall group | P 61 | P 61 |
| Moiety formula | 2(C20 H21 Cl2 F3 N O8 S), 4(C22 H24 Cl2 F3 N O10 S), 2(C2 H3 O2 | 6(C22 H24 Cl2 F3 N O10 S), 3(H2 O) |
| Sum formula | C132 H150 Cl12 F18 N6 O63 S6 | C132 H150 Cl12 F18 N6 O63 S6 |
| Mr | 3788.22 | 3788.23 |
| Dx,g cm-3 | 1.519 | 1.519 |
| Z | 3 | 3 |
| Mu (mm-1) | 3.521 | 3.521 |
| F000 | 5849.8 | 5850.0 |
| F000' | 5889.49 |  |
| h,k,lmax | 28,28,33 | 27,27,33 |
| Nref | 16576[ 8473] | 15162 |
| Tmin,Tmax | 0.400,0.348 | 0.535,0.745 |
| Tmin' | 0.302 |  |

|  |  |
| --- | --- |
| Correction method= # Reported T Limits: Tmin=0.535 Tmax=0.745 AbsCorr = MULTI-SCAN |  |

|  |  |
| --- | --- |
| Data completeness= 1.79/0.91 | Theta(max)= 73.026 |

|  |  |
| --- | --- |
| R(reflections)= 0.0531( 11483) | wR2(reflections)= 0.1498( 15162) |

S = 1.064 | Npar= 1439 |

---

```
The following ALERTS were generated. Each ALERT has the format
       test-name_ALERT_alert-type_alert-level.
Click on the hyperlinks for more details of the test.


---
```

|  |  |
| --- | --- |
|  | **Author Response: This is actually an intramolecular contact.** |

```
---
```

|  |  |
| --- | --- |
|  | **Author Response: This is actually an intramolecular contact.** |

```
PLAT911_ALERT_3_C Missing FCF Refl Between Thmin & STh/L=    0.600         16 Report
PLAT915_ALERT_3_C No Flack x Check Done: Low Friedel Pair Coverage         86 %     


---

Alert level G
PLAT002_ALERT_2_G Number of Distance or Angle Restraints on AtSite         83 Note  
PLAT003_ALERT_2_G Number of Uiso or Uij Restrained non-H Atoms ...         28 Report
PLAT042_ALERT_1_G Calc. and Reported MoietyFormula Strings  Differ     Please Check
PLAT068_ALERT_1_G Reported F000 Differs from Calcd (or Missing)...     Please Check
PLAT083_ALERT_2_G SHELXL Second Parameter in WGHT  Unusually Large       9.17 Why ? 
PLAT152_ALERT_1_G The Supplied and Calc. Volume s.u. Differ by ...          2 Units 
PLAT171_ALERT_4_G The CIF-Embedded .res File Contains EADP Records         13 Report
PLAT172_ALERT_4_G The CIF-Embedded .res File Contains DFIX Records         29 Report
PLAT176_ALERT_4_G The CIF-Embedded .res File Contains SADI Records          4 Report
PLAT178_ALERT_4_G The CIF-Embedded .res File Contains SIMU Records         11 Report
PLAT230_ALERT_2_G Hirshfeld Test Diff for   F26Z     --C28A      .        6.2 s.u.  
PLAT230_ALERT_2_G Hirshfeld Test Diff for   F29A     --C28A      .        5.2 s.u.  
PLAT242_ALERT_2_G Low    'MainMol' Ueq as Compared to Neighbors of       C28B Check 
PLAT242_ALERT_2_G Low    'MainMol' Ueq as Compared to Neighbors of       C28A Check 
PLAT300_ALERT_4_G Atom Site Occupancy of Cl2        Constrained at        0.5 Check

And 83 other PLAT300 Alerts

PLAT300_ALERT_4_G Atom Site Occupancy of Cl3        Constrained at        0.5 Check 
PLAT300_ALERT_4_G Atom Site Occupancy of Cl4        Constrained at        0.5 Check 
PLAT300_ALERT_4_G Atom Site Occupancy of Cl5        Constrained at        0.5 Check 
PLAT300_ALERT_4_G Atom Site Occupancy of F29C       Constrained at        0.5 Check 
PLAT300_ALERT_4_G Atom Site Occupancy of F29D       Constrained at        0.5 Check 
PLAT300_ALERT_4_G Atom Site Occupancy of F30C       Constrained at        0.5 Check 
PLAT300_ALERT_4_G Atom Site Occupancy of F30D       Constrained at        0.5 Check 
PLAT300_ALERT_4_G Atom Site Occupancy of F31C       Constrained at        0.5 Check 
PLAT300_ALERT_4_G Atom Site Occupancy of F31D       Constrained at        0.5 Check 
PLAT300_ALERT_4_G Atom Site Occupancy of O20C       Constrained at        0.5 Check 
PLAT300_ALERT_4_G Atom Site Occupancy of O20D       Constrained at        0.5 Check 
PLAT300_ALERT_4_G Atom Site Occupancy of O22C       Constrained at        0.5 Check 
PLAT300_ALERT_4_G Atom Site Occupancy of O22D       Constrained at        0.5 Check 
PLAT300_ALERT_4_G Atom Site Occupancy of O32C       Constrained at        0.5 Check 
PLAT300_ALERT_4_G Atom Site Occupancy of O32D       Constrained at        0.5 Check 
PLAT300_ALERT_4_G Atom Site Occupancy of O33C       Constrained at        0.5 Check 
PLAT300_ALERT_4_G Atom Site Occupancy of O33D       Constrained at        0.5 Check 
PLAT300_ALERT_4_G Atom Site Occupancy of O34C       Constrained at        0.5 Check 
PLAT300_ALERT_4_G Atom Site Occupancy of O34D       Constrained at        0.5 Check 
PLAT300_ALERT_4_G Atom Site Occupancy of O35C       Constrained at        0.5 Check 
PLAT300_ALERT_4_G Atom Site Occupancy of O35D       Constrained at        0.5 Check 
PLAT300_ALERT_4_G Atom Site Occupancy of O37C       Constrained at        0.5 Check 
PLAT300_ALERT_4_G Atom Site Occupancy of O37D       Constrained at        0.5 Check 
PLAT300_ALERT_4_G Atom Site Occupancy of N25C       Constrained at        0.5 Check 
PLAT300_ALERT_4_G Atom Site Occupancy of N25D       Constrained at        0.5 Check 
PLAT300_ALERT_4_G Atom Site Occupancy of C2C        Constrained at        0.5 Check 
PLAT300_ALERT_4_G Atom Site Occupancy of C2D        Constrained at        0.5 Check 
PLAT300_ALERT_4_G Atom Site Occupancy of C3C        Constrained at        0.5 Check 
PLAT300_ALERT_4_G Atom Site Occupancy of C3D        Constrained at        0.5 Check 
PLAT300_ALERT_4_G Atom Site Occupancy of C4C        Constrained at        0.5 Check 
PLAT300_ALERT_4_G Atom Site Occupancy of C4D        Constrained at        0.5 Check 
PLAT300_ALERT_4_G Atom Site Occupancy of C5C        Constrained at        0.5 Check 
PLAT300_ALERT_4_G Atom Site Occupancy of C5D        Constrained at        0.5 Check 
PLAT300_ALERT_4_G Atom Site Occupancy of C6C        Constrained at        0.5 Check 
PLAT300_ALERT_4_G Atom Site Occupancy of C6D        Constrained at        0.5 Check 
PLAT300_ALERT_4_G Atom Site Occupancy of C7C        Constrained at        0.5 Check 
PLAT300_ALERT_4_G Atom Site Occupancy of C7D        Constrained at        0.5 Check 
PLAT300_ALERT_4_G Atom Site Occupancy of C8C        Constrained at        0.5 Check 
PLAT300_ALERT_4_G Atom Site Occupancy of C8D        Constrained at        0.5 Check 
PLAT300_ALERT_4_G Atom Site Occupancy of C9C        Constrained at        0.5 Check 
PLAT300_ALERT_4_G Atom Site Occupancy of C9D        Constrained at        0.5 Check 
PLAT300_ALERT_4_G Atom Site Occupancy of C21C       Constrained at        0.5 Check 
PLAT300_ALERT_4_G Atom Site Occupancy of C21D       Constrained at        0.5 Check 
PLAT300_ALERT_4_G Atom Site Occupancy of C23C       Constrained at        0.5 Check 
PLAT300_ALERT_4_G Atom Site Occupancy of C23D       Constrained at        0.5 Check 
PLAT300_ALERT_4_G Atom Site Occupancy of C28C       Constrained at        0.5 Check 
PLAT300_ALERT_4_G Atom Site Occupancy of C28D       Constrained at        0.5 Check 
PLAT300_ALERT_4_G Atom Site Occupancy of C36C       Constrained at        0.5 Check 
PLAT300_ALERT_4_G Atom Site Occupancy of C36D       Constrained at        0.5 Check 
PLAT300_ALERT_4_G Atom Site Occupancy of C38C       Constrained at        0.5 Check 
PLAT300_ALERT_4_G Atom Site Occupancy of C38D       Constrained at        0.5 Check 
PLAT300_ALERT_4_G Atom Site Occupancy of H4C        Constrained at        0.5 Check 
PLAT300_ALERT_4_G Atom Site Occupancy of H4D        Constrained at        0.5 Check 
PLAT300_ALERT_4_G Atom Site Occupancy of H5C        Constrained at        0.5 Check 
PLAT300_ALERT_4_G Atom Site Occupancy of H5D        Constrained at        0.5 Check 
PLAT300_ALERT_4_G Atom Site Occupancy of H6C        Constrained at        0.5 Check 
PLAT300_ALERT_4_G Atom Site Occupancy of H6D        Constrained at        0.5 Check 
PLAT300_ALERT_4_G Atom Site Occupancy of H7C        Constrained at        0.5 Check 
PLAT300_ALERT_4_G Atom Site Occupancy of H7D        Constrained at        0.5 Check 
PLAT300_ALERT_4_G Atom Site Occupancy of H8C        Constrained at        0.5 Check 
PLAT300_ALERT_4_G Atom Site Occupancy of H8D        Constrained at        0.5 Check 
PLAT300_ALERT_4_G Atom Site Occupancy of H3CA       Constrained at        0.5 Check 
PLAT300_ALERT_4_G Atom Site Occupancy of H3CB       Constrained at        0.5 Check 
PLAT300_ALERT_4_G Atom Site Occupancy of H9CA       Constrained at        0.5 Check 
PLAT300_ALERT_4_G Atom Site Occupancy of H9CB       Constrained at        0.5 Check 
PLAT300_ALERT_4_G Atom Site Occupancy of H3DA       Constrained at        0.5 Check 
PLAT300_ALERT_4_G Atom Site Occupancy of H3DB       Constrained at        0.5 Check 
PLAT300_ALERT_4_G Atom Site Occupancy of H9DA       Constrained at        0.5 Check 
PLAT300_ALERT_4_G Atom Site Occupancy of H9DB       Constrained at        0.5 Check 
PLAT300_ALERT_4_G Atom Site Occupancy of H23A       Constrained at        0.5 Check 
PLAT300_ALERT_4_G Atom Site Occupancy of H23B       Constrained at        0.5 Check 
PLAT300_ALERT_4_G Atom Site Occupancy of H23C       Constrained at        0.5 Check 
PLAT300_ALERT_4_G Atom Site Occupancy of H23D       Constrained at        0.5 Check 
PLAT300_ALERT_4_G Atom Site Occupancy of H25C       Constrained at        0.5 Check 
PLAT300_ALERT_4_G Atom Site Occupancy of H25D       Constrained at        0.5 Check 
PLAT300_ALERT_4_G Atom Site Occupancy of H33C       Constrained at        0.5 Check 
PLAT300_ALERT_4_G Atom Site Occupancy of H33D       Constrained at        0.5 Check 
PLAT300_ALERT_4_G Atom Site Occupancy of H38A       Constrained at        0.5 Check 
PLAT300_ALERT_4_G Atom Site Occupancy of H38B       Constrained at        0.5 Check 
PLAT300_ALERT_4_G Atom Site Occupancy of H38C       Constrained at        0.5 Check 
PLAT300_ALERT_4_G Atom Site Occupancy of H38D       Constrained at        0.5 Check 
PLAT300_ALERT_4_G Atom Site Occupancy of H2WA       Constrained at        0.5 Check 
PLAT300_ALERT_4_G Atom Site Occupancy of H2WB       Constrained at        0.5 Check

PLAT301_ALERT_3_G Main Residue  Disorder ..............(Resd  1  )        77% Note

And 2 other PLAT301 Alerts

PLAT301_ALERT_3_G Main Residue  Disorder ..............(Resd  2  )        21% Note  
PLAT301_ALERT_3_G Main Residue  Disorder ..............(Resd  3  )        10% Note

PLAT302_ALERT_4_G Anion/Solvent/Minor-Residue Disorder (Resd  4  )       100% Note

And 4 other PLAT302 Alerts

PLAT302_ALERT_4_G Anion/Solvent/Minor-Residue Disorder (Resd  5  )       100% Note  
PLAT302_ALERT_4_G Anion/Solvent/Minor-Residue Disorder (Resd  6  )       100% Note  
PLAT302_ALERT_4_G Anion/Solvent/Minor-Residue Disorder (Resd  8  )       100% Note  
PLAT302_ALERT_4_G Anion/Solvent/Minor-Residue Disorder (Resd  9  )       100% Note

PLAT304_ALERT_4_G Non-Integer Number of Atoms in ...... Resd  4          2.28 Check

And 6 other PLAT304 Alerts

PLAT304_ALERT_4_G Non-Integer Number of Atoms in ...... Resd  5          3.21 Check 
PLAT304_ALERT_4_G Non-Integer Number of Atoms in ...... Resd  6          1.51 Check 
PLAT304_ALERT_4_G Non-Integer Number of Atoms in ...... Resd  8          0.10 Check 
PLAT304_ALERT_4_G Non-Integer Number of Atoms in ...... Resd  9          0.40 Check 
PLAT304_ALERT_4_G Non-Integer Number of Atoms in ...... Resd 10          0.50 Check 
PLAT304_ALERT_4_G Non-Integer Number of Atoms in ...... Resd 11          0.50 Check

PLAT311_ALERT_2_G Isolated Disordered Oxygen Atom (No H's ?) .....        O1W Check 
PLAT311_ALERT_2_G Isolated Disordered Oxygen Atom (No H's ?) .....        O2W Check 
PLAT432_ALERT_2_G Short Inter X...Y Contact  S13C     ..C1Z              2.44 Ang.

And 10 other PLAT432 Alerts

PLAT432_ALERT_2_G Short Inter X...Y Contact  S13C     ..C1C              2.81 Ang.  
PLAT432_ALERT_2_G Short Inter X...Y Contact  S13C     ..C1D              2.83 Ang.  
PLAT432_ALERT_2_G Short Inter X...Y Contact  F30B     ..C36A             2.89 Ang.  
PLAT432_ALERT_2_G Short Inter X...Y Contact  O37C     ..C21B             2.88 Ang.  
PLAT432_ALERT_2_G Short Inter X...Y Contact  O37D     ..C21B             2.82 Ang.  
PLAT432_ALERT_2_G Short Inter X...Y Contact  O10D     ..C15C             2.95 Ang.  
PLAT432_ALERT_2_G Short Inter X...Y Contact  C14C     ..C1Z              2.91 Ang.  
PLAT432_ALERT_2_G Short Inter X...Y Contact  C14C     ..C1D              3.04 Ang.  
PLAT432_ALERT_2_G Short Inter X...Y Contact  C14C     ..C1C              3.16 Ang.  
PLAT432_ALERT_2_G Short Inter X...Y Contact  C15C     ..C1D              3.11 Ang.

PLAT720_ALERT_4_G Number of Unusual/Non-Standard Labels ..........         20 Note  
PLAT790_ALERT_4_G Centre of Gravity not Within Unit Cell: Resd.  #          2 Note  
              C22 H24 Cl2 F3 N O10 S                                            
PLAT790_ALERT_4_G Centre of Gravity not Within Unit Cell: Resd.  #          3 Note  
              C22 H24 Cl2 F3 N O10 S                                            
PLAT790_ALERT_4_G Centre of Gravity not Within Unit Cell: Resd.  #          4 Note  
              C2 H3 O2                                                          
PLAT790_ALERT_4_G Centre of Gravity not Within Unit Cell: Resd.  #          5 Note  
              C2 H3 O2                                                          
PLAT790_ALERT_4_G Centre of Gravity not Within Unit Cell: Resd.  #          6 Note  
              C2 H3 O2                                                          
PLAT790_ALERT_4_G Centre of Gravity not Within Unit Cell: Resd.  #          7 Note  
              H2 O                                                              
PLAT790_ALERT_4_G Centre of Gravity not Within Unit Cell: Resd.  #          8 Note  
              O                                                                 
PLAT790_ALERT_4_G Centre of Gravity not Within Unit Cell: Resd.  #          9 Note  
              O                                                                 
PLAT790_ALERT_4_G Centre of Gravity not Within Unit Cell: Resd.  #         10 Note  
              H                                                                 
PLAT790_ALERT_4_G Centre of Gravity not Within Unit Cell: Resd.  #         11 Note  
              H                                                                 
PLAT791_ALERT_4_G Model has Chirality at C2A         (Chiral SPGR)          R Verify

And 21 other PLAT791 Alerts

PLAT791_ALERT_4_G Model has Chirality at C2B         (Chiral SPGR)          R Verify
PLAT791_ALERT_4_G Model has Chirality at C4A         (Chiral SPGR)          S Verify
PLAT791_ALERT_4_G Model has Chirality at C4B         (Chiral SPGR)          S Verify
PLAT791_ALERT_4_G Model has Chirality at C4C         (Chiral SPGR)          S Verify
PLAT791_ALERT_4_G Model has Chirality at C4D         (Chiral SPGR)          S Verify
PLAT791_ALERT_4_G Model has Chirality at C5A         (Chiral SPGR)          R Verify
PLAT791_ALERT_4_G Model has Chirality at C5B         (Chiral SPGR)          R Verify
PLAT791_ALERT_4_G Model has Chirality at C5C         (Chiral SPGR)          R Verify
PLAT791_ALERT_4_G Model has Chirality at C5D         (Chiral SPGR)          R Verify
PLAT791_ALERT_4_G Model has Chirality at C6A         (Chiral SPGR)          R Verify
PLAT791_ALERT_4_G Model has Chirality at C6B         (Chiral SPGR)          R Verify
PLAT791_ALERT_4_G Model has Chirality at C6C         (Chiral SPGR)          R Verify
PLAT791_ALERT_4_G Model has Chirality at C6D         (Chiral SPGR)          R Verify
PLAT791_ALERT_4_G Model has Chirality at C7A         (Chiral SPGR)          R Verify
PLAT791_ALERT_4_G Model has Chirality at C7B         (Chiral SPGR)          R Verify
PLAT791_ALERT_4_G Model has Chirality at C7C         (Chiral SPGR)          R Verify
PLAT791_ALERT_4_G Model has Chirality at C7D         (Chiral SPGR)          R Verify
PLAT791_ALERT_4_G Model has Chirality at C8A         (Chiral SPGR)          R Verify
PLAT791_ALERT_4_G Model has Chirality at C8B         (Chiral SPGR)          R Verify
PLAT791_ALERT_4_G Model has Chirality at C8C         (Chiral SPGR)          R Verify
PLAT791_ALERT_4_G Model has Chirality at C8D         (Chiral SPGR)          R Verify

PLAT811_ALERT_5_G No ADDSYM Analysis: Too Many Excluded Atoms ....          ! Info 
PLAT860_ALERT_3_G Number of Least-Squares Restraints .............        437 Note  
PLAT912_ALERT_4_G Missing # of FCF Reflections Above STh/L=  0.600        193 Note  
PLAT978_ALERT_2_G Number C-C Bonds with Positive Residual Density.          1 Info  


---

   1 ALERT level A = Most likely a serious problem - resolve or explain
   1 ALERT level B = A potentially serious problem, consider carefully
  20 ALERT level C = Check. Ensure it is not caused by an omission or oversight
 163 ALERT level G = General information/check it is not something unexpected

   3 ALERT type 1 CIF construction/syntax error, inconsistent or missing data
  35 ALERT type 2 Indicator that the structure model may be wrong or deficient
  10 ALERT type 3 Indicator that the structure quality may be low
 136 ALERT type 4 Improvement, methodology, query or suggestion
   1 ALERT type 5 Informative message, check
```

---

---

It is advisable to attempt to resolve as many as possible of the alerts in all categories. Often the minor alerts point to easily fixed oversights, errors and omissions in your CIF or refinement strategy, so attention to these fine details can be worthwhile. In order to resolve some of the more serious problems it may be necessary to carry out additional measurements or structure refinements. However, the purpose of your study may justify the reported deviations and the more serious of these should normally be commented upon in the discussion or experimental section of a paper or in the "special\_details" fields of the CIF. checkCIF was carefully designed to identify outliers and unusual parameters, but every test has its limitations and alerts that are not important in a particular case may appear. Conversely, the absence of alerts does not guarantee there are no aspects of the results needing attention. It is up to the individual to critically assess their own results and, if necessary, seek expert advice. **Publication of your CIF in IUCr journals** A basic structural check has been run on your CIF. These basic checks will be run on all CIFs submitted for publication in IUCr journals (*Acta Crystallographica*, *Journal of Applied Crystallography*, *Journal of Synchrotron Radiation*); however, if you intend to submit to *Acta Crystallographica Section C* or *E* or *IUCrData*, you should make sure that full publication checks are run on the final version of your CIF prior to submission. **Publication of your CIF in other journals** Please refer to the *Notes for Authors* of the relevant journal for any special instructions relating to CIF submission. |

---

**PLATON version of 23/04/2018; check.def file version of 23/04/2018**

|  |
| --- |
| **Datablock sads\_h** - ellipsoid plot |
|  |

---

 Download CIF editor (publCIF) from the IUCr   
 Download CIF editor (enCIFer) from the CCDC   
 Test a new CIF entry 
