# Supplementary material for: Comparison of glycosyl donors: a supramer approach
Source: Beilstein J Org Chem. 2024 Jan 31;20:181–92. doi: 10.3762/bjoc.20.18 (PMC10840533; doi:10.3762/bjoc.20.18)
Supplement: File 2 — Deposed crystallographic information file (CCDC 1843708) and check file. [file Beilstein_J_Org_Chem-20-181-s002.zip › checkCIF_PLATON report_PDF.pdf]

No syntax errors found.
Please wait while processing ....

CIF dictionary
Interpreting this report

Datablock: sads_h

Bond precision:	C-C = 0.0124 A		Wavelength=1.54178
Cell:	a=22.9311(10)	b=22.9311(10)	c=27.2831(12)
	alpha=90	beta=90	gamma=120
Temperature:	120 K		

	Calculated	Reported
Volume	12424.4(14)	12424.4(12)
Space group	P 61	P 61
Hall group	P 61	P 61
Moiety formula	2(C20 H21 Cl2 F3 N O8 S), 4(C22 H24 Cl2 F3 N O10 S), 2(C2 H3 O2	6(C22 H24 Cl2 F3 N O10 S), 3(H2 O)
Sum formula	C132 H150 Cl12 F18 N6 O63 S6	C132 H150 Cl12 F18 N6 O63 S6
Mr	3788.22	3788.23
Dx,g cm-3	1.519	1.519
Z	3	3
Mu (mm-1)	3.521	3.521
F000	5849.8	5850.0
F000'	5889.49	
h,k,lmax	28,28,33	27,27,33
Nref	16576[8473]	15162
Tmin,Tmax	0.400,0.348	0.535,0.745
Tmin'	0.302	

Correction method= # Reported T Limits: Tmin=0.535 Tmax=0.745
AbsCorr = MULTI-SCAN

Data completeness= 1.79/0.91 Theta(max)= 73.026

R(reflections)= 0.0531(11483) wR2(reflections)= 0.1498(15162)

S = 1.064 Npar= 1439

The following ALERTS were generated. Each ALERT has the format
test-name_ALERT_alert-type_alert-level.
Click on the hyperlinks for more details of the test.

Alert level A

PLAT430_ALERT_2_A	Short Inter D...A Contact S13C	..O11Z	2.78	Ang.

Author Response: This is actually an intramolecular contact.

Alert level B

PLAT340_ALERT_3_B	Low Bond Precision on C-C Bonds	0.01235	Ang.

Alert level C

PLAT089_ALERT_3_C	Poor Data / Parameter Ratio (Zmax < 18)	5.71	Note
PLAT215_ALERT_3_C	Disordered Cl4 has ADP max/min Ratio	3.3	Note
PLAT220_ALERT_2_C	Non-Solvent Resd 1 C Ueq(max)/Ueq(min) Range	3.5	Ratio
PLAT222_ALERT_3_C	Non-Solv. Resd 1 H Uiso(max)/Uiso(min) Range	10.0	Ratio
PLAT234_ALERT_4_C	Large Hirshfeld Difference S13C --C2D	0.16	Ang.
PLAT234_ALERT_4_C	Large Hirshfeld Difference F29B --C28B	0.20	Ang.
PLAT242_ALERT_2_C	Low 'MainMol' Ueq as Compared to Neighbors of	C14C	Check
PLAT245_ALERT_2_C	U(iso) H25C Smaller than U(eq) N25C by	0.031	Ang**2

And 3 other PLAT245 Alerts
More ...

PLAT331_ALERT_2_C	Small Average Phenyl C-C Dist C14C -C19C	1.37	Ang.
PLAT411_ALERT_2_C	Short Inter H...H Contact H18C ..H16Z	2.11	Ang.
PLAT413_ALERT_2_C	Short Inter XH3 .. XHn H12H ..H12C	2.07	Ang.

And 2 other PLAT413 Alerts
More ...

PLAT414_ALERT_2_C	Short Intra D-H..H-X H8D ..H34C	1.91	Ang.
PLAT430_ALERT_2_C	Short Inter D...A Contact O22B ..O1W	2.88	Ang.

Author Response: This is actually an intramolecular contact.

PLAT911_ALERT_3_C	Missing FCF Refl Between Thmin & STh/L= 0.600	16	Report
PLAT915_ALERT_3_C	No Flack x Check Done: Low Friedel Pair Coverage	86	%

Alert level G

PLAT002_ALERT_2_G	Number of Distance or Angle Restraints on AtSite	83	Note

```
PLAT003_ALERT_2_G Number of Uiso or Uij Restrained non-H Atoms ...         28 Report
PLAT042_ALERT_1_G Calc. and Reported MoietyFormula Strings  Differ     Please Check
PLAT068_ALERT_1_G Reported F000 Differs from Calcd (or Missing)...     Please Check
PLAT083_ALERT_2_G SHELXL Second Parameter in WGHT  Unusually Large       9.17 Why ?
PLAT152_ALERT_1_G The Supplied and Calc. Volume s.u. Differ by ...          2 Units
PLAT171_ALERT_4_G The CIF-Embedded .res File Contains EADP Records         13 Report
PLAT172_ALERT_4_G The CIF-Embedded .res File Contains DFIX Records         29 Report
PLAT176_ALERT_4_G The CIF-Embedded .res File Contains SADI Records          4 Report
PLAT178_ALERT_4_G The CIF-Embedded .res File Contains SIMU Records         11 Report
PLAT230_ALERT_2_G Hirshfeld Test Diff for    F26Z      --C28A      .      6.2 s.u.
PLAT230_ALERT_2_G Hirshfeld Test Diff for    F29A      --C28A      .      5.2 s.u.
PLAT242_ALERT_2_G Low    'MainMol' Ueq as Compared to Neighbors of       C28B Check
PLAT242_ALERT_2_G Low    'MainMol' Ueq as Compared to Neighbors of       C28A Check
PLAT300_ALERT_4_G Atom Site Occupancy of Cl2        Constrained at        0.5 Check
```

And 83 other PLAT300 Alerts

More ...

```
PLAT301_ALERT_3_G Main Residue  Disorder .............(Resd  1  )         77% Note
```

And 2 other PLAT301 Alerts

More ...

```
PLAT302_ALERT_4_G Anion/Solvent/Minor-Residue Disorder (Resd  4  )       100% Note
```

And 4 other PLAT302 Alerts

More ...

```
PLAT304_ALERT_4_G Non-Integer Number of Atoms in ...... Resd  4          2.28 Check
```

And 6 other PLAT304 Alerts

More ...

```
PLAT311_ALERT_2_G Isolated Disordered Oxygen Atom (No H's ?) .....        O1W Check
PLAT311_ALERT_2_G Isolated Disordered Oxygen Atom (No H's ?) .....        O2W Check
PLAT432_ALERT_2_G Short Inter X...Y Contact  S13C     ..C1Z              2.44 Ang.
```

And 10 other PLAT432 Alerts

More ...

```
PLAT720_ALERT_4_G Number of Unusual/Non-Standard Labels ..........         20 Note
PLAT790_ALERT_4_G Centre of Gravity not Within Unit Cell: Resd.  #          2 Note
              C22 H24 Cl2 F3 N O10 S
PLAT790_ALERT_4_G Centre of Gravity not Within Unit Cell: Resd.  #          3 Note
              C22 H24 Cl2 F3 N O10 S
PLAT790_ALERT_4_G Centre of Gravity not Within Unit Cell: Resd.  #          4 Note
              C2 H3 O2
PLAT790_ALERT_4_G Centre of Gravity not Within Unit Cell: Resd.  #          5 Note
              C2 H3 O2
PLAT790_ALERT_4_G Centre of Gravity not Within Unit Cell: Resd.  #          6 Note
              C2 H3 O2
PLAT790_ALERT_4_G Centre of Gravity not Within Unit Cell: Resd.  #          7 Note
              H2 O
PLAT790_ALERT_4_G Centre of Gravity not Within Unit Cell: Resd.  #          8 Note
              O
PLAT790_ALERT_4_G Centre of Gravity not Within Unit Cell: Resd.  #          9 Note
              O
PLAT790_ALERT_4_G Centre of Gravity not Within Unit Cell: Resd.  #         10 Note
              H
PLAT790_ALERT_4_G Centre of Gravity not Within Unit Cell: Resd.  #         11 Note
              H
PLAT791_ALERT_4_G Model has Chirality at C2A        (Chiral SPGR)           R Verify
```

And 21 other PLAT791 Alerts

More ...

```
PLAT811_ALERT_5_G No ADDSYM Analysis: Too Many Excluded Atoms ....          ! Info
PLAT860_ALERT_3_G Number of Least-Squares Restraints .............        437 Note
PLAT912_ALERT_4_G Missing # of FCF Reflections Above STh/L=  0.600        193 Note
PLAT978_ALERT_2_G Number C-C Bonds with Positive Residual Density.          1 Info
```

```
  1 ALERT level A = Most likely a serious problem - resolve or explain
  1 ALERT level B = A potentially serious problem, consider carefully
 20 ALERT level C = Check. Ensure it is not caused by an omission or oversight
163 ALERT level G = General information/check it is not something unexpected

  3 ALERT type 1 CIF construction/syntax error, inconsistent or missing data
 35 ALERT type 2 Indicator that the structure model may be wrong or deficient
 10 ALERT type 3 Indicator that the structure quality may be low
136 ALERT type 4 Improvement, methodology, query or suggestion
  1 ALERT type 5 Informative message, check
```

It is advisable to attempt to resolve as many as possible of the alerts in all categories. Often the minor alerts point to easily fixed oversights, errors and omissions in your CIF or refinement strategy, so attention to these fine details can be worthwhile. In order to resolve some of the more serious problems it may be necessary to carry out additional measurements or structure refinements. However, the purpose of your study may justify the reported deviations and the more serious of these should

normally be commented upon in the discussion or experimental section of a paper or in the "special_details" fields of the CIF. checkCIF was carefully designed to identify outliers and unusual parameters, but every test has its limitations and alerts that are not important in a particular case may appear. Conversely, the absence of alerts does not guarantee there are no aspects of the results needing attention. It is up to the individual to critically assess their own results and, if necessary, seek expert advice.

Publication of your CIF in IUCr journals

A basic structural check has been run on your CIF. These basic checks will be run on all CIFs submitted for publication in IUCr journals (*Acta Crystallographica*, *Journal of Applied Crystallography*, *Journal of Synchrotron Radiation*); however, if you intend to submit to *Acta Crystallographica Section C* or *E* or *IUCrData*, you should make sure that full publication checks are run on the final version of your CIF prior to submission.

Publication of your CIF in other journals

Please refer to the *Notes for Authors* of the relevant journal for any special instructions relating to CIF submission.

PLATON version of 23/04/2018; check.def file version of 23/04/2018

Datablock sads_h - ellipsoid plot

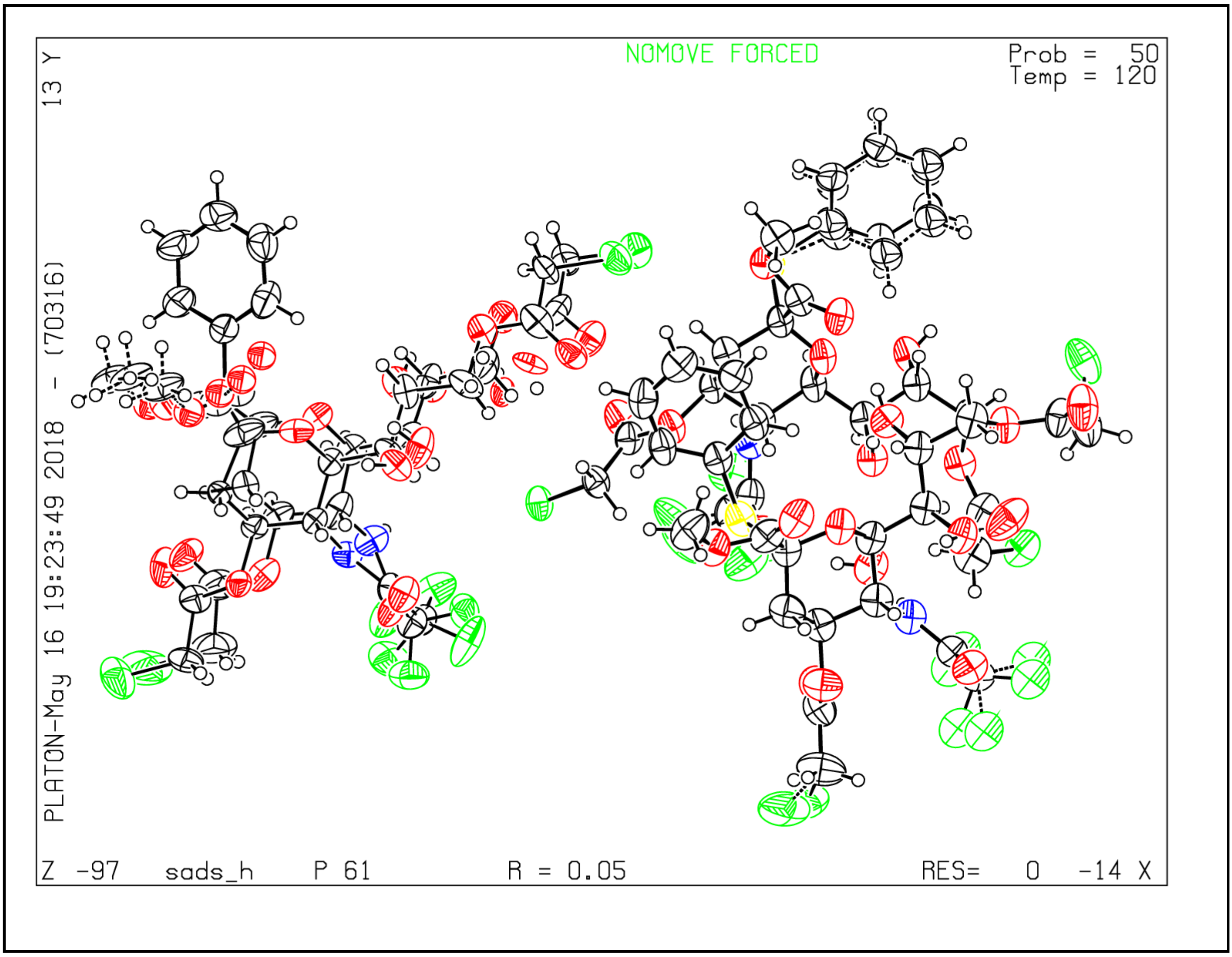

Download CIF editor (publCIF) from the IUCr
Download CIF editor (enCIFer) from the CCDC
Test a new CIF entry
